# Comparative genomics reveals modular virulence repertoires and extensive horizontal gene transfer in Vibrio species associated with white syndrome of Porites cylindrica

**DOI:** 10.1099/mgen.0.001862

**Published:** 2026-10-23

**Authors:** Ewelina Rubin, Gaurav Shimpi, Héloïse Rouzé, Bastian Bentlage

**Affiliations:** 1 Marine Laboratory, University of Guam, Mangilao, GU 96923, Guam

**Keywords:** corals, microbiome, white syndrome

## Abstract

Coral white syndrome (WS) is a widespread condition characterized by tissue loss and skeletal exposure, with multiple bacterial pathogens – particularly *Vibrio* species – implicated in its aetiology. To investigate the genomic basis of potential virulence in *Vibrio* associated with WS of *Porites cylindrica*, we isolated 57 *Vibrio* strains from healthy and diseased coral tissues collected from reefs in Guam. Thirteen representative strains from five dominant species *– Vibrio coralliilyticus*, *Vibrio owensii*, *Vibrio tubiashii*, *Vibrio harveyi* and *Vibrio tetraodonis* – were selected for whole-genome sequencing using Oxford Nanopore Technology. Comparative genomic analyses revealed a conserved repertoire of extracellular enzymes, including haemolysins, cytolysins, metalloproteases and subtilisin-like peptidases, alongside lineage-specific toxin and regulatory modules. Variation in secretion systems (T1SS–T6SS), particularly in T3SS, T4SS and T6SS subtypes, reflected diversification in host interactions and competitive capacities across species. Mobile genetic elements, including plasmids and prophages, contributed additional virulence-associated genes and secretion clusters, underscoring the role of horizontal gene transfer in shaping the accessory genome content. Notably, *V. coralliilyticus* harboured homologues of the cholera toxin-associated genes *ace* and *zot*, although their relatively low aa similarity to characterized *Vibrio cholerae* toxins suggests that their functional roles remain uncertain. Together, these comparative genomic analyses suggest that virulence-associated gene repertoires are broadly distributed among coral-associated *Vibrio* and are organized as a conserved ecological core complemented by flexible, horizontally acquired accessory modules. These findings provide hypotheses regarding the genomic basis of opportunistic pathogenicity that will require future functional validation. This study provides the first comparative genomic framework for *Vibrio* associated with *P. cylindrica*, providing a foundation for future investigations into how genomic plasticity and modular virulence gene repertoires may contribute to opportunistic disease dynamics in coral reef ecosystems.

## Data Summary

All genome sequence data generated in this study have been deposited in the NCBI database under BioProject accession number PRJNA1354586, with associated BioSample accessions SAMN52948606–SAMN52948618. All supporting data required to reproduce the analyses presented in this study are included within the manuscript and its Supplementary Material. Additional data files, including genome annotations and comparative genomics outputs, are available from the corresponding author upon reasonable request.

**Impact Statement**

Coral diseases are increasing globally and threaten the persistence of reef ecosystems, yet the microbial mechanisms underlying these diseases remain poorly understood. This study uses comparative genomics to investigate *Vibrio* strains associated with white syndrome in the coral *Porites cylindrica*. We show that virulence potential is not restricted to a single pathogen but is instead distributed across multiple lineages and shaped by a flexible pool of horizontally acquired genes, including plasmids and prophages. These findings support an opportunistic disease model in which environmental conditions and microbial community dynamics influence disease outcomes. By providing a genomic framework for understanding coral-associated *Vibrio*, this work contributes to the development of molecular tools for monitoring and diagnosing coral disease and offers broader insights into how horizontal gene transfer drives the evolution of pathogenicity in marine bacteria.

## Introduction

*Porites cylindrica* is one of the dominant reef-building corals throughout the Indo-Pacific [1, 2]. It is considered a highly resilient species, capable of recovering from both bleaching events and disease outbreaks [1, 3, 4]. In Guam, Micronesia, *P. cylindrica* accounts for up to 73% of coral cover across surveyed reefs [5, 6]. Despite its ecological importance and resilience, this species is chronically affected by white syndrome (WS) lesions [7–9]. The causes of WS in *P. cylindrica* remain unknown. In other coral species, however, WS has been associated with multiple aetiologies [10, 11]. Consequently, WS is regarded not as a single disease but as a disease complex characterized by a common clinical sign – progressive tissue loss that exposes the underlying white coral skeleton [12, 13]. This tissue loss may result from environmental stress, infectious agents or interactions between the two and can lead to partial or complete colony mortality in both natural reefs and coral restoration projects [11, 13]. Following tissue loss, the exposed skeleton is rapidly colonized by other organisms, most commonly turf algae and cyanobacteria [14]. Further research is needed to identify the microorganisms associated with *P. cylindrica* WS, understand the mechanisms underlying lesion development and progression and determine the causal pathways involved. As seawater temperatures continue to rise and anthropogenic pressures intensify, coral disease outbreaks are expected to become more frequent and severe [15]. Improving our understanding of coral disease aetiology and progression will be essential for developing effective diagnostic tools, monitoring strategies and future interventions to support coral reef conservation.

Bacterial pathogens are among the most frequently investigated potential causes of coral WSs. For example, WS in *Pocillopora damicornis c*an be triggered by opportunistic infection with *Vibrio coralliilyticus* strain BAA-450 during periods of thermal stress, when elevated seawater temperatures suppress host immunity [10]. WS lesions in the same coral species have also been associated with *Vibrio harveyi* [16]. Similarly, six *Vibrio* isolates have been identified as primary pathogens associated with WS in three Indo-Pacific coral species (*Pachyseris speciosa*, *Montipora aequituberculata* and *Acropora cytherea*) [17]. One of these isolates was subsequently identified through whole-genome sequencing as *V. coralliilyticus* strain P1 [18]. Additional *Vibrio* strains associated with WS include *V. coralliilyticus* strain OCN008, which causes acute WS in *Montipora capitata* [19], and *Vibrio owensii* strain OCN002, which causes a chronic form of the disease in the same host [20]. *Vibrio tubiashii* has also been implicated in WS of *Porites lutea* [14], whereas *Vibrio alginolyticus* has been associated with WS in *Porites andrewsi* [21]. Collectively, these studies indicate that multiple *Vibrio* species have been linked to WS across diverse coral hosts. Consistent with these observations, a previous microbiome study of *P. cylindrica* from Guam detected the genus *Vibrio* in 58% of WS lesions collected from two reef sites [22]. However, the genomic diversity and evolutionary relationships of the *Vibrio* lineages associated with these lesions remain unknown.

The genus *Vibrio* is large and taxonomically diverse, comprising nearly 150 described species that are abundant in estuarine and marine environments. In addition to coral pathogens, the genus includes species that cause disease in humans, such as *Vibrio cholerae*, *Vibrio vulnificus* and *Vibrio parahaemolyticus*, as well as pathogens of fish (e.g. *V. harveyi*, *V. alginolyticus* and *Vibrio ponticus*), crustaceans (e.g. *V. owensii*) and molluscs (e.g. *V. coralliilyticus*) [23–30]. Owing to their ecological, clinical and economic importance, the molecular mechanisms underlying *Vibrio* pathogenicity have been studied extensively [31–34].

Many *Vibrio* strains harbour a diverse repertoire of virulence-associated factors that facilitate host recognition, colonization and persistence. However, pathogenicity is not a universal trait within the genus, and even closely related strains can differ markedly in their virulence potential. In many cases, virulence-associated genes are acquired through horizontal gene transfer (HGT). A well-known example is the acquisition of the cholera toxin (*ctx*) genes by pathogenic *V. cholerae* through infection with the CTXφ bacteriophage [24].

Advances in whole-genome sequencing and comparative genomics have greatly expanded our understanding of *Vibrio* evolution, adaptation and pathogenicity. Comparisons among strains with different ecological niches or pathogenic phenotypes can identify candidate virulence-associated genes, horizontally acquired genomic regions and other genetic features that generate hypotheses regarding host interactions. For example, comparative genomic analysis of two *V. coralliilyticus* strains (P1 and BAA-450) identified differences in pathogenicity islands and virulence-associated genes [35]. Similarly, comparison of four *V. coralliilyticus* strains pathogenic to both corals and oyster larvae suggested that prophage-mediated HGT contributes to the dissemination of virulence-associated genes within *Vibrio* populations [36]. A broader comparative analysis of more than 20 *Vibrio* genomes demonstrated that chitin metabolism is a conserved feature of the genus, while expansion of chitinase gene families in some species reflects adaptation to chitin-rich environments [37]. Likewise, comparative genomic analysis of a shrimp-pathogenic *Vibrio* strain identified a plasmid carrying the toxin genes responsible for acute hepatopancreatic necrosis disease (AHPND) [38]. Collectively, these studies demonstrate the value of comparative genomics for identifying genomic features associated with pathogenicity and adaptation while generating hypotheses for future functional investigation.

Despite the frequent association of *Vibrio* species with coral WS across multiple coral hosts, the genomic diversity and modular virulence architecture of *Vibrio* strains associated with WS lesions in *P. cylindrica* remain poorly understood. In particular, it is unclear how virulence-associated genes, secretion systems and horizontally acquired elements are distributed among strains isolated from healthy and diseased coral tissues, or whether these genomic features are restricted to specific lineages or broadly distributed across coral-associated *Vibrio* populations. Here, we use comparative genomics to analyse representative strains from the five dominant *Vibrio* species isolated from healthy and WS-affected *P. cylindrica* colonies collected from reefs in Guam. Including isolates from both healthy and diseased corals allowed us to compare the genomic diversity of *Vibrio* populations across contrasting host health states while maximizing the genetic diversity represented in our dataset. Because many *Vibrio* species are recognized as opportunistic rather than obligate pathogens, isolates recovered from healthy coral tissues were included as genomic background controls rather than presumed non-pathogenic strains. By characterizing virulence-associated gene repertoires, secretion system architectures and mobile genetic elements (MGEs), we aim to identify conserved and lineage-specific genomic features that may influence host interactions. This comparative genomic framework provides a foundation for generating testable hypotheses regarding the potential role of *Vibrio* in WS pathogenesis and how HGT and genomic plasticity may shape coral disease dynamics under changing environmental conditions.

## Methods

### Bacterial isolation

Seventeen coral samples, each ~2 cm in length, were collected from ten colonies of *P. cylindrica* at two shallow (1–2 m depth) reef flat sites in Guam: Tumon Bay and Luminao. At Tumon Bay, all four sampled colonies displayed WS lesions. From each colony, one sample was taken from healthy tissue and one from diseased tissue. In contrast, at Luminao, three of the six sampled colonies exhibited WS lesions, while the other three appeared healthy. For the colonies with WS, both healthy and diseased tissues were collected, whereas only healthy tissues were sampled from the disease-free colonies. Coral branches were cut using sterilized wire cutters, which were cleaned between samples by immersion in 10% bleach followed by 95% ethanol. The samples were placed in sterile plastic bags and kept on ice during transport to the Marine Laboratory at the University of Guam. In the lab, each coral branch was rinsed with 0.2-µm-filtered, sterile water to limit seawater-associated surface bacteria. The coral tissue was then scraped off using sterilized scalpels and tweezers. The resulting tissue slurry was transferred into sterile microcentrifuge tubes and streaked onto culture plates for bacterial culture. To maximize the diversity of cultivated bacteria, four different types of culture media were used: (1) marine agar (MA), (2) peptone seawater media (PEP), (3) tryptone seawater media (TRY) and (4) thiosulphate citrate bile salts agar (TCBS). MA was prepared using Marine Broth 2216 (Millipore) and agar (Wako) according to the manufacturer’s protocols. TCBS (BD) was prepared based on the manufacturer’s protocol. The PEP medium consisted of 3 g of Bacto™ proteose peptone (BD), 2 g of Bacto™ yeast extract (Difco) and 15 g of agar (Wako) per litre of 0.2 µm-filtered, sterilized seawater. The TRY medium consisted of 3 g Bacto™ tryptone (BD), 2 g Bacto™ yeast extract (Difco) and 15 g agar (Wako) per litre of 0.2 µm-filtered, sterilized seawater. Each coral tissue slurry was streaked in duplicate on all four media types. From each plate, between three and five single bacterial colonies were picked and subcultured onto media plates at least three times to ensure the isolation of pure, axenic cultures. Liquid cultures of the isolated bacteria were prepared in the same medium without agar; they were preserved as glycerol stocks at a final glycerol concentration of 15% and stored at –80 °C.

### Bacterial identification

Liquid cultures, each 3 ml, were grown for 12 to 18 h at 28 °C. Following incubation, bacterial cells were pelleted by centrifugation, and DNA was extracted from the pellets using the GenCatch Blood and Tissue Genomic Mini Prep Kit (Epoch Life Science). The 16S rRNA gene was amplified using the universal bacterial primers 27F and 1492R under the PCR cycling conditions described by Galkiewicz and Kellogg [39]. PCR amplifications were performed using GoTaq Flexi Taq Polymerase and reagents according to the manufacturer’s protocol for a 25 ul reaction (Promega, Madison, WI). The PCR cycling conditions were as follows: an initial denaturation step for 2 min at 95 °C, followed by 34 cycles of 95 °C for 45 s, 50 °C for 45 s and 72 °C for 90 s, with a final extension of 5 min at 72 °C. For all isolates identified as *Vibrio* (*n*=57), a second PCR amplification targeting the RNA polymerase gene (*rpoA*) was performed to obtain species-level identification. The *rpoA*-PCR was performed using the primers and PCR conditions described by Thompson *et al*. [40]. PCR amplifications for the *rpoA* gene were performed using GoTaq® Flexi Taq Polymerase and reagents following the manufacturer’s protocol for a 25 ul reaction (Promega, Madison, WI), and the PCR cycling conditions were as follows: an initial denaturation step for 2 min at 95 °C, followed by 30 cycles of 95 °C for 30 s, 50 °C for 30 s and 72 °C for 60 s, with a final extension of 5 min at 72 °C. The resulting PCR products were purified using the GeneJET PCR Purification Kit (Thermo Fisher, Vilnius, Lithuania) and sent to Epoch Life Science Inc. services (Missouri City, TX) for Sanger sequencing. The obtained sequences were trimmed for quality control using Geneious software, and genus-level taxonomic identification was performed using 16S sequences in blast searches against the 16S rRNA Silva database v138.2 [41], with 100% pairwise nt identity and 100% query coverage. For *Vibrio* isolates (*n*=57), the *rpoA* gene was used in a blast search against the NCBI nt core nt database, and species identification was determined using 100% query coverage and ≥99.5% nt identity.

The final species-level identifications of the 13 strains selected for genome sequencing were confirmed using average nucleotide identity (ANI) calculated with FastANI [42] and genome-scale phylogenetic analysis. The 13 genomes generated in this study were analysed together with 175 *Vibrio* reference genomes and one *Aliivibrio fischeri* outgroup genome downloaded from NCBI (File S1, available in the online Supplementary Material) using GTDB-Tk v2.7.2 [43]. Genomes were processed using the identify, align and classify workflows, and the GTDB-Tk bacterial marker set (bac120) was used to generate a concatenated aa alignment of 120 conserved bacterial marker genes. Maximum-likelihood phylogenetic inference was performed from the bac120 alignment using RAxML v8.2.12 [44] under the PROTGAMMAILGF model, corresponding to the Le and Gascuel (LG) aa substitution matrix with empirical aa frequencies, a gamma model of among-site rate heterogeneity and an estimated proportion of invariant sites. Branch support was assessed using 100 rapid bootstrap replicates with the RAxML -f a algorithm, which performs rapid bootstrapping and a search for the best-scoring ML tree in a single run. The resulting phylogeny was visualized and annotated using Interactive Tree Of Life (iTOL) [45], with bootstrap support values ≥80% displayed (Fig. S1).

### Whole-genome sequencing

A total of 13 *Vibrio* strains were selected for whole-genome sequencing. Selection was performed in two stages. First, representative isolates were chosen from the five most frequently recovered *Vibrio* species identified among the 57 *Vibrio* isolates (Table 1). Second, within each species, isolates were selected to maximize representation of both reef sites (Tumon and Luminao) and, whenever available, both healthy and diseased coral tissue. When multiple isolates met these criteria, strains were selected at random. Glycerol stocks of the selected strains were cultured in the original isolation media. Bacterial cells were then pelleted by centrifugation, and high-molecular-weight DNA was extracted using the Monarch HMW DNA Extraction Kit (New England Biolabs, Ipswich, MA). Sequencing libraries were prepared using the Ligation Sequencing Kit and the Native Barcoding Kit 24 from Oxford Nanopore Technologies (Oxfordshire, UK). Sequencing was performed on an R10 Nanopore flow cell (FLO-MIN114) paired with kit 14 chemistry on a MinION device over a 48-h run time. The raw signal data (fast5 files) were base-called using Guppy basecaller v2.2.3 (Oxford Nanopore Technologies) with the super-accuracy base-calling model in MinKNOW (version: 24.02.16).

**Table 1.** Number of *Vibrio* strains per species isolated from healthy (H) and diseased (D) tissue of *P. cylindrica* colonies originating from two reef sites: Tumon (TU) and Luminao (LU) Species assignments were based on *rpoA* gene sequencing of all 57 *Vibrio* isolates.

	Number of isolates	TU-D	LU-D	TU-H	LU-H
*V. owensii*	11	2	4	4	1
*V. tubiashii*	11	10	0	1	0
*Vibrio tetraodonis/aquimaris*	11	0	10	1	0
*V. coralliilyticus*	10	7	2	0	1
*V. harveyi*	5	4	0	1	0
*Vibrio maritimus*	4	1	3	0	0
*V. ponticus*	1	0	0	0	1
*Vibrio ishigakensis*	1	0	0	0	1
*Vibrio neptunius*	1	1	0	0	0
*Vibrio* spp.	2	0	1	1	0
Total	57	25	20	8	4

### Genome assembly and annotation

Genome assemblies were generated using Flye v2.9.5 with the flag- -nano-hq and all the rest set to default settings [46], and the completeness of the assemblies was evaluated using BUSCO [47], with the reference lineage set to vibrio_odb12. Genome annotations were obtained using the Rapid Annotation using Subsystem Technology (RAST) server [48]. The annotated protein-coding sequences, provided in FASTA format by the RAST server, were analysed using VFanalyzer [49] to identify virulence factor homologues shared with other pathogenic *Vibrio* species. These protein sequences were also submitted to the BlastKOALA server [50] to obtain KEGG identifiers for functional annotation. Protein sequences annotated by RAST as peptidases or proteases were further analysed by comparing them with the MEROPS peptidase database [51], using local blast searches. Peptidases were assigned to the MEROPS family with a cutoff >40% aa identity using a minimum of 70% query coverage.

To predict horizontally transferred genomic regions, IslandViewer4 [52] and AlienHunter [53] were employed. The latter tool was run on the Proksee genome visualization platform [54]. Because IslandViewer4 and AlienHunter employ fundamentally different algorithms for HGT prediction and can produce substantially different predictions, their outputs were used to identify candidate HGT regions rather than to define HGT events based solely on overlapping predictions. Instead, predicted regions containing virulence-associated genes or secretion system loci were independently evaluated by whole-chromosome blast alignments against closely related reference genomes. This comparative approach was used to assess whether predicted HGT regions corresponded to strain-specific insertions absent from closely related genomes. The whole-chromosome sequence comparisons were performed using the blast ring alignment functionality implemented in the Proksee genome visualization platform [54]. For each chromosome and plasmid, the assembled genome was used as a reference sequence and compared with closely related *Vibrio* genomes retrieved from NCBI, using ANI (>95% when available). blastn alignments were generated using default Proksee parameters, and regions lacking significant nt similarity to reference genomes were identified as candidate strain-specific insertions. Bacteriophage sequences were identified using the online PHASTER tool [55]. To assess plasmid-mediated HGT, plasmid sequences were queried against the NCBI database using blast to identify homologous plasmids. We selected plasmid hits that met the following criteria (≥85% nt identity and >20% query coverage) and used these plasmids to conduct ANI analysis. Finally, plasmids with ANI >85% were aligned and visualized using the Prokseek tool. Secretion system components identified in the *Vibrio* genomes were grouped according to the KEGG BRITE classification system (available at https://www.kegg.jp/brite/ko02044). This system enabled us to classify secretion-associated proteins into six major secretion systems and their respective subtypes. Specifically, the type II secretion system (T2SS) was further divided into four subsystems: the general secretion pathway (T2SS-GSP), the secretion and assembly of mannose-sensitive haemagglutinin pili (T2SS-MSHA), the secretion and assembly of other pili or fimbriae (T2SS-PILIN) and the secretion and assembly of tight adherence pili (T2SS-TAD). Similarly, the type III secretion system was subdivided into components related to flagellar assembly (T3SS-FLA) and those related to secretion systems distinct from flagella (T3SS). The type IV secretion system was divided into three categories: two associated with the assembly of conjugative pili required for plasmid transfer (T4SS-CON-P and T4SS-TRB) and one related to the transfer of DNA and proteins not associated with plasmid DNA (T4SS-DNA-PR). Figures showing gene arrangements in secretion systems were constructed using the Proksee tool or the gggenes package [56] in R v4.5.1.

## Results

### *Vibrio* isolates and genome characteristics

A total of 115 bacterial strains were isolated from both healthy and diseased tissues of *P. cylindrica*, including representatives of *Gammaproteobacteria*, *Alphaproteobacteria*, *Bacteroidia* and *Actinobacteria* (Table S1). Among the 115 bacterial isolates recovered, 57 (49%) were identified as members of the genus *Vibrio* based on 16S rRNA gene sequencing and were subsequently subjected to rpoA gene sequencing for species-level identification. The most frequently isolated *Vibrio* species were *V. owensii* (11 isolates), *V. tubiashii* (11 isolates), members of the *Vibrio aquimaris/tetraodonis* clade (11 isolates with 99% nt identity of the *rpoA* gene between the two species), *V. coralliilyticus* (10 isolates) and *V. harveyi* (5 isolates) (Table 1). *V. tubiashii* was recovered exclusively from corals originating from Tumon (Table 1).

Of the 57 *Vibrio* isolates, 41 were obtained from diseased coral tissue and 16 from visually healthy tissue. Representative strains from the five most frequently isolated *Vibrio* species were selected for whole-genome sequencing. Selection aimed to maximize representation of both reef sites and coral health states whenever possible (Table 1). The genome assemblies of the 13 sequenced strains were highly complete, with BUSCO scores exceeding 99% and sequencing coverage ranging from 52× to 148× (Table 2). The final species-level identifications of the 13 sequenced strains were supported by genome-scale phylogenetic analysis and ANI calculated using FastANI v1.34 [42] with default parameters. In the phylogenomic analysis based on the GTDB-Tk bac120 marker set [43], all 13 strains clustered with reference genomes corresponding to their respective species assignments (Fig. S1). ANI values ranged from 95.19 to 98.54%, exceeding the widely accepted ≥95% species delineation threshold for all strains (Table S3). The isolates LU3DMA2 and LU5DMA4 clustered with *V. tetraodonis* in the phylogenomic tree and were most closely related to the *V. tetraodonis* type strain based on ANI (95.19–95.47%), whereas their ANI values relative to *V. aquimaris* were below 95% (Table S3). Reference genomes and accession numbers used for the ANI comparisons are provided in Table S3.

**Table 2.** Genome characteristics of 13 *Vibrio* strains belonging to 5 species isolated in this study The strain label indicates (1) location (LU or TU), (2) sample number, (3) tissue type (e.g. 5D, sample number 5 diseased tissue; 4H, sample number 4 healthy tissue) and (4) isolation media (e.g. MA, PEP and TRY).

Species	Strain	Tissue type	Site	Genome length	Mean coverage	No. of chromosomes	No. of plasmids	BUSCO score (%)
*Vibrio tetraodonis*	LU5DMA4	D	LU	4,761,502	78	1	0	98.2
	LU3DMA2	D	LU	4,542,275	92	2	0	98.0
*V. coralliilyticus*	TU5DMA1	D	TU	5,796,936	102	2	1	99.5
	LU3DPEP4	D	LU	5,777,417	148	2	1	99.7
*V. harveyi*	TU3DPEP3	D	TU	5,798,052	52	2	0	99.1
	TU4HPEP4	H	TU	5,805,957	138	2	0	99.2
*V. owensii*	TU8HTRY5	H	TU	5,977,422	118	2	0	99.5
	TU3DMA2	D	TU	6,461,482	87	2	1	99.2
	LU5DTRY1	D	LU	6,100,886	121	2	1	99.2
	LU8HTRY1	H	LU	5,943,102	89	2	0	98.1
*V. tubiashii*	TU3DMA1	D	TU	5,684,442	104	2	2	99.5
	TU1DMA1	D	TU	5,318,319	145	2	1	99.7
	TU1DMA2	D	TU	5,423,814	162	2	1	99.6

Twelve of the 13 sequenced strains exhibited the typical bipartite *Vibrio* genome organization consisting of 2 chromosomes. The larger chromosomes ranged from 3.26 to 3.80 Mbp, and the smaller chromosomes ranged from 1.27 to 2.47 Mbp (Table 2). In contrast, strain LU5DMA4 (*V. tetraodonis*) possessed a single chromosome of ~4.76 Mbp (Table 2). Six strains harboured plasmids. Two strains each of *V. coralliilyticus*, *V. owensii* and *V. tubiashii* carried a single plasmid, and one *V. tubiashii* strain contained two plasmids (Table 2). The remaining strains lacked plasmids.

### Virulence-associated genes

Comparative genomic analyses were performed to assess the distribution of previously characterized *Vibrio* virulence-associated genes across 13 strains isolated from healthy and diseased *P. cylindrica* tissues. In total, 17 distinct virulence-associated gene categories were identified (Fig. 1). These genes were classified as (i) lineage-restricted or strain-specific or (ii) broadly distributed across most or all strains.

**Fig. 1.**
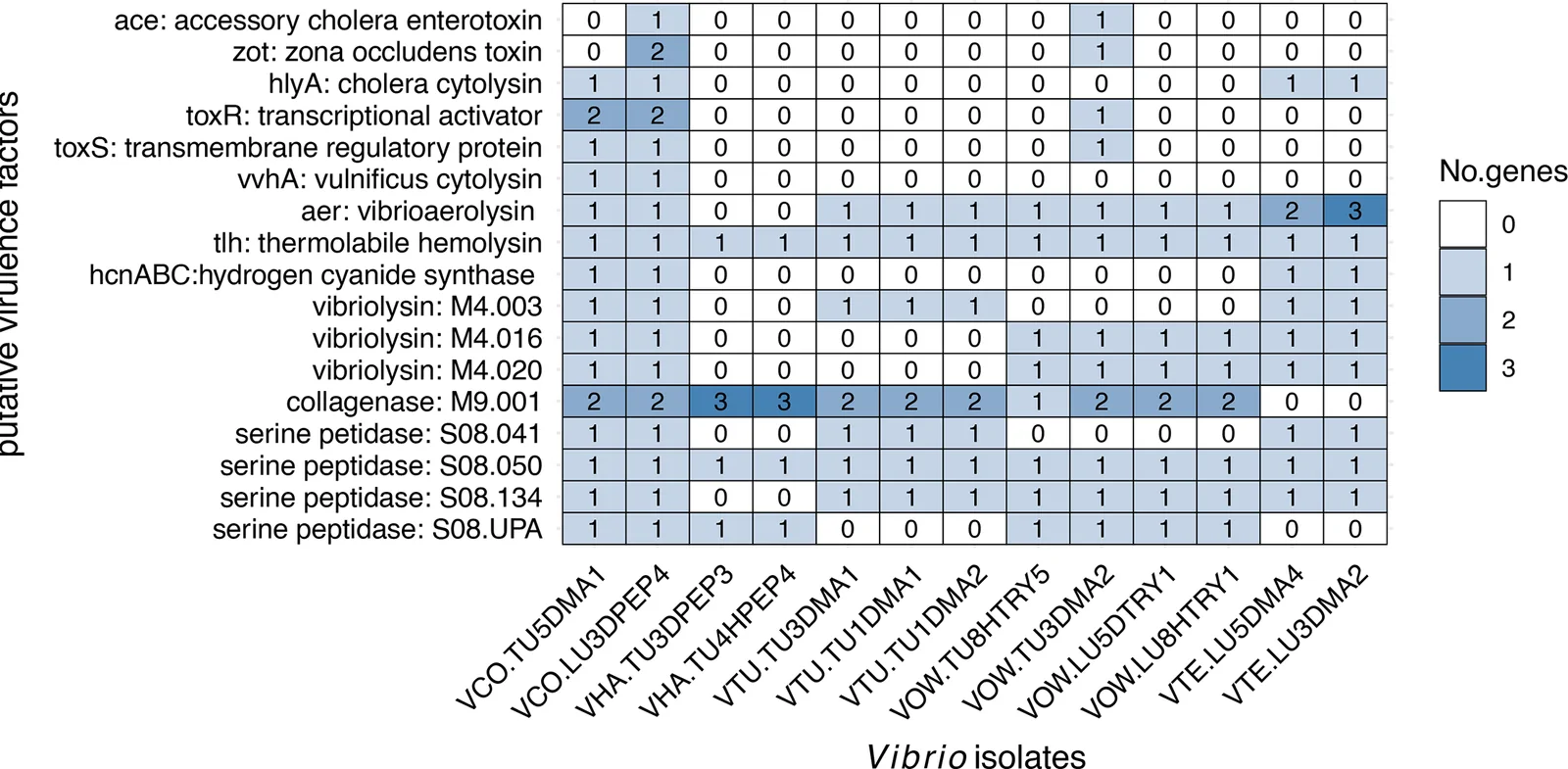
Distribution and copy number of predicted virulence-associated genes across 13 *Vibrio* strains isolated from healthy and WS lesion tissues of *P. cylindrica*. Heatmap values indicate the number of predicted homologues per genome (0–3 copies). Strains are grouped by species: *V. coralliilyticus* (VCO), *V. harveyi* (VHA), *V. tubiashii* (VTU), *V. owensii* (VOW) and *V. tetraodonis* (VTE). Putative virulence-associated genes were identified using the Virulence Factors Database (VFDB) and VFanalyzer, which predicts orthologous relationships to curated virulence factors based on sequence similarity and genomic context. Absence (0) indicates no detectable homologue under the applied search criteria.

#### Lineage-restricted and strain-specific virulence genes

Homologues of the *V. cholerae* cytolysin *hlyA* were identified in *V. coralliilyticus* and *V. tetraodonis*. These homologues shared 40–49% aa identity with *V. cholerae hlyA* but lacked the beta-prism lectin domain (Fig. S3A). A homologue of the *V. vulnificus* haemolysin *vvhA* was detected exclusively in *V. coralliilyticus* strains, and those showed 76% aa identity to *V. vulnificus* (Figs 1 and S2B). Other homologues of cholera-associated genes, including *ace*, *zot*, *toxR* and *toxS*, were also detected (Figs 1 and S3). The *ace* homologue in strain VCO.LU3DPEP4 showed 57% aa identity to *V. cholerae* KWW43308, whereas the *ace*-like gene in strain VOW.TU3DMA2 showed 25% aa identity (Fig. S3C). Two genes containing the zonular occludent domain (*zot*) were present in the *V. coralliilyticus* strain LU3DPEP4 and showed 53% aa identity to *V. cholerae* protein WP_000021609 and 68% identity to WP_275082003 (Figs 1 and S3D). The *hcnABC* gene cluster, encoding hydrogen cyanide synthases, was identified only in *V. coralliilyticus* and *V. tetraodonis* strains, with protein identities ranging from 42 to 61% to the hcnABC proteins of *Pseudomonas aeruginosa* PAO1 (Figs 1 and S3E). Collectively, these findings identify homologues of several virulence-associated genes based on sequence similarity; however, sequence homology alone does not establish functional conservation. Consequently, the biological roles of these proteins in coral-associated *Vibrio* remain to be experimentally verified through gene expression studies, functional characterization and infection assays.

#### Common and broadly distributed virulence factors

Several extracellular proteases were broadly distributed across multiple taxa (Fig. 1). These included Vibrio aerolysins, metalloproteases (vibriolysins), collagenases and subtilisins. M4.003-type vibriolysins were detected in *V. coralliilyticus*, *V. tubiashii* and *V. tetraodonis*, whereas related M4.016 and M4.020 subtypes were identified in *V. coralliilyticus*, *V. tetraodonis* and *V. owensii* (Fig. 1). Collagenases (M9.001 subfamily) were present in most species, except *V. tetraodonis* and *V. harveyi,* which harboured three distinct collagenase genes (Fig. 1). Subtilisin-type serine proteases (MEROPS S8 family) were identified in all strains except strains of *V. harveyi*, including S08.134-like homologues of *vvp1*/vvsa/asp (Fig. 1). Additional subtilase subtypes (S08.41, S08.50 and S08.UPA) were also detected and predicted to contain signal peptides, indicating secretion (Fig. 1). S08.50-like subtilases were present in all strains and showed similarity to *vhp1* from *V. harveyi*. The phospholipase-type cytolysin (*tlh*) was universally present across all 13 strains (Fig. 1).

### Distribution and diversity of secretion systems

Comparative genomic analysis of 13 coral-associated *Vibrio* strains identified multiple secretion systems typical of Gram-negative bacteria, including T1SS-T6SS (Figs 2, 3 and 4). All strains encoded gene clusters required for flagellar assembly (T3SS-FLA). However, the number and organization of clusters varied among species (Fig. 2). *V. harveyi* and *V. owensii* strains possessed two flagellar gene clusters: one encoding polar flagella (~32 genes) on the large chromosome and a second encoding lateral flagella (26–29 genes) on the smaller chromosome (Fig. 2). All three *V. tubiashii* strains contained two gene clusters (27+4 genes), both located on the large chromosome. The two *V. coralliilyticus* strains differed in the number of clusters (three and four, respectively). In addition to polar flagellar clusters (26+4 genes), both strains contained 41-gene clusters consistent with lateral flagellar systems. One *V. coralliilyticus* strain (LU3DPEP4) harboured an additional 23-gene flagellar assembly cluster within a predicted HGT region; based on gene count, this cluster appears incomplete. All strains encoded conserved T2SS-MSHA and T2SS-PILIN systems (Fig. 2). In contrast, T2SS-TAD systems showed substantial variability. A total of 36 T2SS-TAD gene clusters were identified across 13 genomes, ranging from one to five per strain (Fig. 2b). The highest copy number (five TAD clusters) was observed in two *V. tubiashii* strains, whereas one *V. owensii* strain contained a single TAD cluster (Fig. 2b). Canonical T3SS injectosome clusters were identified in six strains belonging to *V. coralliilyticus*, *V. tetraodonis* and *V. harveyi* (Figs 2 and 3). The T3SS clusters in *V. coralliilyticus* and *V. tetraodonis* resembled T3SS3, containing homologues of SseBCD translocation proteins and Salmonella-associated effectors (Fig. 3, classification after [57, 57]). In contrast, *V. harveyi* strains harboured T3SS clusters similar to *Yersinia*-like T3SS1 systems (Fig. 3). Multiple T4SS subtypes were identified, frequently associated with plasmids (Fig. 2). The T4SS-CON-P system was detected on plasmids in four strains and encoded genes consistent with conjugative pilus formation. A T4SS-TRB operon was identified on plasmid pVOW-LU5DTRY1-63 in *V. owensii*. Additionally, a T4SS-DNA-PR subtype was identified in four strains (two *V. harveyi* and two *V. owensii*). All strains encoded T6SS clusters, although the number of subtypes varied by lineage (Fig. 4). *V. owensii* and *V. harveyi* strains possessed three T6SS subtypes, whereas *V. coralliilyticus*, *V. tetraodonis* and *V. tubiashii* strains harboured two. Four structural T6SS subtypes (named after the first and last gene) were identified: (1) TssM–VgrG (present in all strains), (2) TssL–VasH (present in 11 strains), (3) PpkA–PAAR (restricted to *V. owensii* and *V. harveyi*) and (4) VasL–PAAR (restricted to two *V. tubiashii* strains) (Fig. 4). The TssM–VgrG subtype was typically located on chromosome 1, while TssL–VasH was located on chromosome 2. Searches for nine previously described anti-eukaryotic T6SS2 effectors identified eight of nine effectors in *V. coralliilyticus* strains. Homologues of several effectors were also detected in *V. tubiashii* and *V. tetraodonis* strains.

**Fig. 2.**
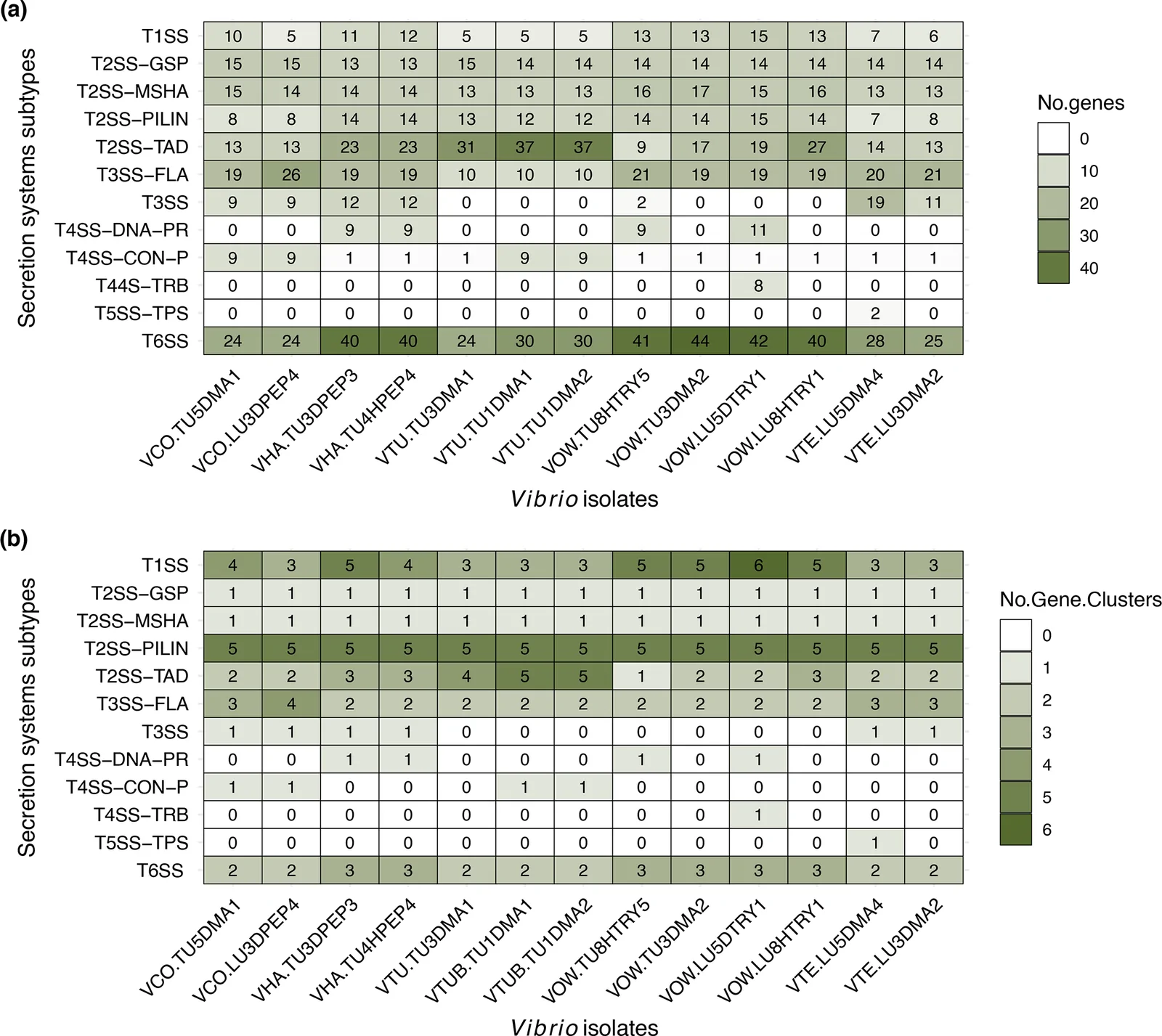
Distribution and organization of secretion system subtypes across 13 *Vibrio* strains isolated from *P. cylindrica*. (**a**) Total number of annotated genes assigned to each secretion system subtype based on KEGG BRITE classification (ko02044). (**b**) Number of discrete gene clusters identified per secretion system subtype within each genome. Secretion systems were grouped into 12 categories: type I secretion system (T1SS); type II secretion systems – general secretion pathway (T2SS-GSP), MSHA pilus biogenesis (T2SS-MSHA), pilin secretion and assembly proteins (T2SS-PILIN) and tight adherence export apparatus (T2SS-TAD); type III secretion systems – flagellar export apparatus (T3SS-FLA) and non-flagellar T3SS; type IV secretion systems – DNA and protein transfer (T4SS-DNA-PR), conjugative pilus assembly (T4SS-CON-P) and TRB system (T4SS-TRB); type V secretion system – two-partner secretion (T5SS-TPS); and type VI secretion system (T6SS). Strains are grouped by species: *V. coralliilyticus* (VCO), *V. harveyi* (VHA), *V. tubiashii* (VTU), *V. owensii* (VOW) and *V. tetraodonis* (VTE).

**Fig. 3.**
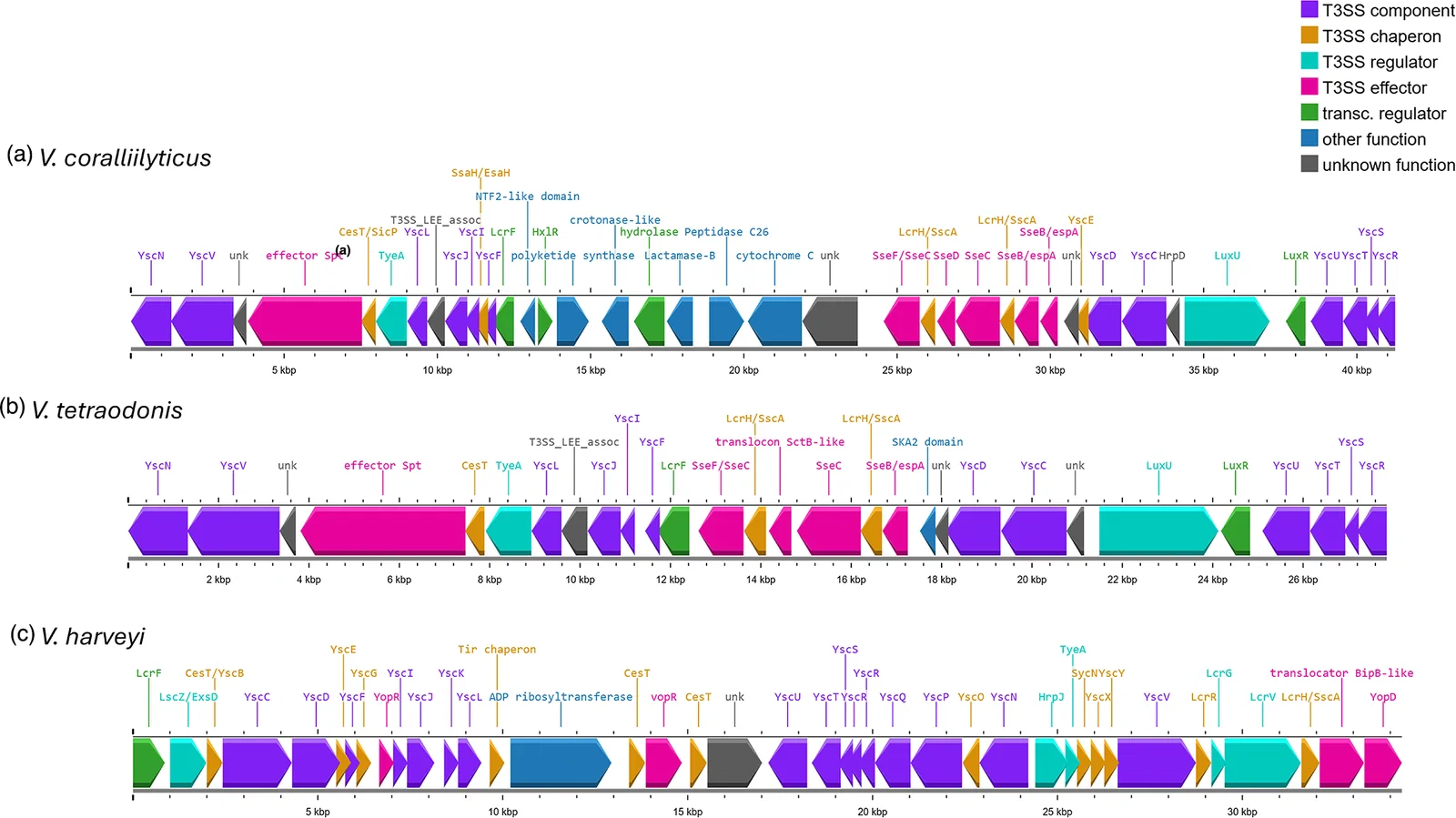
Comparative organization of type III secretion system (T3SS) gene clusters in three *Vibrio* species isolated from *P. cylindrica*. (**a**) *V. coralliilyticus*, (**b**) *V. tetraodonis* and (**c**) *V. harveyi*. Arrows represent predicted ORFs and indicate gene orientation. Colours denote functional categories: T3SS structural components (purple), chaperones (orange), regulators (green), predicted effectors (pink), transcriptional regulators (teal), other annotated functions (blue) and unknown/hypothetical proteins (grey). Gene names are shown above the arrows. Scale bars indicate genomic distance (kb). Synteny and gene-content differences among species highlight the diversification of T3SS architecture across coral-associated *Vibrio* lineages.

**Fig. 4.**
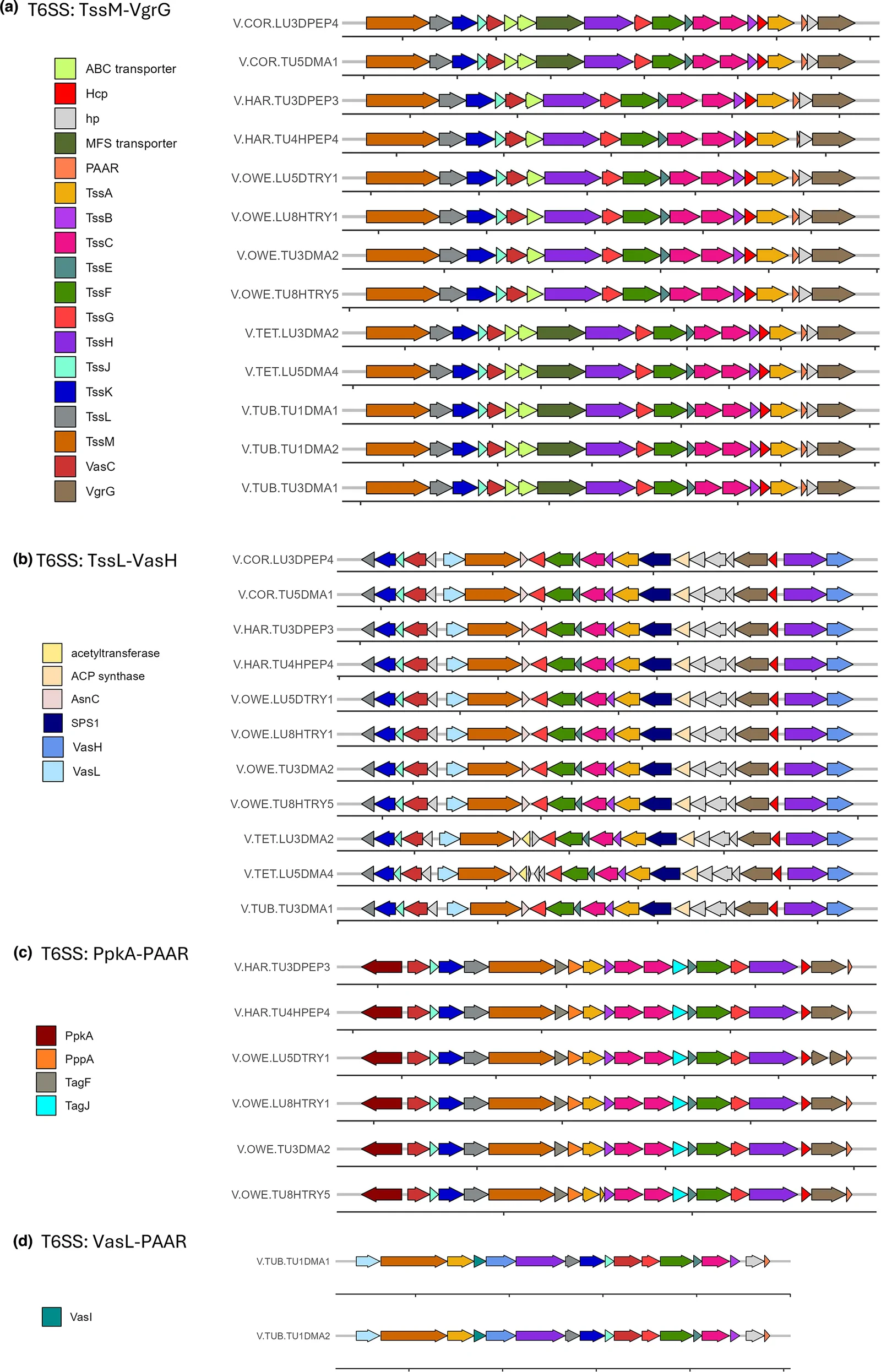
Comparative organization of type VI secretion system (T6SS) subtypes across coral-associated *Vibrio* strains isolated from *P. cylindrica*. Gene cluster architectures are shown for four structural T6SS subtypes: (**a**) TssM–VgrG, (**b**) TssL–VasH, (**c**) PpkA–PAAR and (**d**) VasL–PAAR. Arrows represent predicted ORFs and indicate gene orientation. Colours correspond to core structural components and accessory proteins as indicated in the legends for each panel. Scale bars represent genomic distance (kb). Strains are grouped by species: *V. coralliilyticus* (V.COR), *V. harveyi* (V.HAR), *V. tubiashii* (V.TUB), *V. owensii* (V.OWE) and *V. tetraodonis* (V.TET). Conserved core structural genes are present across multiple species, whereas subtype-specific modules and accessory components vary among lineages.

### HGT elements in coral-associated *Vibrio* genomes

Horizontally acquired elements were identified across thirteen *Vibrio* genomes, including (i) plasmids (Table 3), (ii) prophages and episomal phages (Table 4) and (iii) genomic islands (GIs) and other MGEs predicted by IslandViewer4 and AlienHunter (Table 5). All detected plasmids and prophages were associated with strains isolated from diseased coral tissues, whereas GIs were present in both healthy- and disease-derived isolates.

**Table 3.** Comparative analysis of plasmids from *Vibrio* species associated with *P. cylindrica* and other marine invertebrates Plasmid size, NCBI accession numbers and ANI relative to reference plasmids are shown. Plasmids sequenced in this study are indicated in bold. Plasmids are grouped according to ANI-based similarity to reference plasmids and correspond to comparative clusters visualized in Figs 5, 6 and S5. Reference plasmids used for each comparison are indicated by 100% ANI values (see figure references in the ANI column).

Species	Strain	Host species	Plasmid name	Length (bp)	NCBI accession	ANI (%)
*V. coralliilyticus*	**LU3DPEP4**	*P. cylindrica*	**pVCO-LU3DPEP4-384**	383,902		100 (Fig. 5 reference)
	**TU5DMA1**	*P. cylindrica*	**pVCO-TU5DMA1-460**	459,903		98.34
	RE98	Shellfish hatchery	pVCO-RE98-319	319,400	CP009620.1	98.11
	Rb102	*Strongylocentrotus intermedius*	pVCO-Rb102-324	324,189	CP120721.1	97.49
	OCN014	*A. cytherea*	pVCO-OCN014-381	380,781	CP009266.2	96.75
	RE22	*Crassostrea gigas*	pVCO-RE22-337	337,275	CP031474.1	96.59
	BAA450	*P. damicornis*	pVCO-BAA450-350	397,488	CP156658.1	95.89
	OCN008	*M. capitata*	pVCO-OCN008-245	244,689	CP048695.1	93.94
	S2052	Sediment	pVCO-S2052-224	223,859	CP063053.1	92.45
	SNUTY1	*Crassostrea gigas*	pVCO-SNUTY1-255	254,703	CP020455.1	92.43
	58	*Crassostrea gigas*	pVCO-S58-52	52,449	CP016558.1	84.25
	RE98	Shellfish hatchery	pVCO-RE98-381	380,714	CP009619.1	<80
	SCSIO 43001	*Galaxea fascicularis*	pVCO-SCSIO43001-180	179,815	CP024629.1	<80
	SNUTY1	*Crassostrea gigas*	pVCO-SNUTY1-136	136,423	CP020456.1	<80
*V. parahaemolyticus*	3HP	*Penaeus vannamei*	pVPA-3HP-70	70,452	KP324996	100 (Fig. 6 reference)
*V. owensii*	SH14	*Penaeus vannamei*	pVOW-SH14-69	69,148	CP045861.1	99.43
	V180403	*Penaeus vannamei*	pVOW-V180403-74	74,553	CP033146.1	99.35
	GL-605	*Penaeus vannamei*	pVOW-GL605-77	77,278	CP097860.1	98.08
	1700302	*Penaeus vannamei*	pVOW-1700302-69	68,834	CP033140.1	97.93
	**LU5DTRY1**	*P. cylindrica*	**pVOW-LU5DTRY1-63**	63,222		95.08
	SH14	*Penaeus vannamei*	pVOW-SH14-79	78,918	CP045862.1	93.32
	051011B	Seawater	pVOW-051011B-97	96,819	CP025798.1	93.20
	V180403	*Penaeus vannamei*	pVOW-V180403-73	73,661	CP033147.1	91.79
	GL-605	*Penaeus vannamei*	pVOW-GL605-75	75,054	CP097861.1	90.88
*V. owensii*	**TU3DMA2**	*P. cylindrica*	**pVOW-TU3DMA2-192**	191,976		100.00
*Vibrio campbellii*	Mt009_b1	Seawater	pVCA-Mt009_b1-150	150,486	OX415234.1	88.56
	BF5_0283	Seawater	pVCA-BF5_0283-150	150,483	OX411214.1	87.11
*V. tubiashii*	**TU1DMA2**	*P. cylindrica*	**pVTU-TU1DMA2-71**	71,298		100 (Fig. S5 reference)
	**TU1DMA1**	*P. cylindrica*	**pVTU-TU1DMA1-47**	47,160		94.52
	ATCC 19109	*Mercenaria mercenaria*	pVTU-ATCC19109-57	57,076	CP009358.1	91.53
*V. tubiashii*	**TU3DMA1**	*P. cylindrica*	**pVTU-TU3DMA1-74**	73,988		no NCBI matches
	**TU3DMA1**	*P. cylindrica*	**pVTU-TU3DMA1-350**	350,341		no NCBI matches

**Table 4.** Genomic characteristics of intact prophages and episomal phages identified in *Vibrio* genomes using PHASTER Phage location (ch, chromosome; p, plasmid), predicted genome length, PHASTER score (>90 intact, 70–90=questionable, <70=incomplete) and number of predicted proteins are shown.

Species	Strain	Phage location	Phage name	Phage length (kb)	PHASTER score	No. of proteins
*V. coralliilyticus*	LU3DPEP4	p384 : 238548-2508780	VCO.LU3DPEP4.p384.ph-12.3	12.3	136	20
*V. owensii*	TU3DMA2	ch2 : 2175727-2296339	VOW.TU3DMA2.ch2.ph1-120.6	120.6	140	121
*Vibrio tetraodonis*	LU3DMA2	ch1 : 2688905-2705315	VAQ.LU3DMA2.ch1.ph1-16.4	16.4	110	24
	LU5DMA4	ch1 : 4221842-4238366	VAQ.LU5DMA4.ch1.ph1-16.5	16.5	110	23
	LU5DMA4	ch1 : 3863947-3899017	VAQ.LU5DMA4.ch1.ph2-35.0	35.0	120	49
*V. tubiashii*	TU1DMA2	ch1 : 3443868-3471160	VTU.TU1DMA2.ch1.ph1-27.2	27.2	100	35
	TU1DMA2	ch1 : 1522352-1555632	VTU.TU1DMA2.ch1.ph2-33.2	33.2	150	40
	TU3DMA1	ch1 : 291652-328233	VTU.TU3DMA1.ch1.ph1-36.5	36.5	110	51
	TU3DMA1	ch1 : 600700-637305	VTU.TU3DMA1.ch1.ph2-36.6	36.6	140	48
	TU3DMA1	ch2 : 1263463-1303480	VTU.TU3DMA1.ch2.ph3-40.0	40.0	130	48
	TU3DMA1	p74 : 26-73851	VTU.TU3DMA1.p74.ph4-73.8	73.8	150	105

**Table 5.** Distribution of virulence factors and secretion system genes within predicted and comparatively supported HGT regions HGT-P: regions predicted as horizontally transferred using IslandViewer4 and AlienHunter. HGT-CS: predicted HGT regions with comparative support based on whole-chromosome blast alignments showing absence or discontinuity relative to closely related reference genomes (see Methods). Only HGT regions containing virulence factors or secretion system genes were evaluated for comparative support.

	*V. tubiashii*			*V. owensii*				*V. harveyi*		*V. coralliilyticus*		*V. tetraodonis*	
	TU3DMA1	TU1DMA1	TU1DMA2	TU8HTRY5	TU3DMA2	LU5DTRY1	LU8HTRY1	TU3DPEP3	TU4HPEP4	TU5DMA1	LU3DPEP4	LU5DMA4	LU3DMA2
Virulence factors (total)	8	8	8	7	11	8	8	6	6	14	17	11	12
Virulence factors (HGT-P)	1	2	4	1	3	2	2	1	1	5	1	7	4
Virulence factors (HGT-CS)	1	1	1	0	1	1	1	0	0	1	4	0	0
T1SS (total)	3	3	3	5	5	6	5	5	4	4	3	3	3
T1SS (HGT-P)	2	2	2	3	2	4	3	2	2	3	1	2	2
T1SS (HGT-CS)	1	1	1	1	0	1	1	1	0	1	0	0	2
T2SS-TAD (total)	4	5	5	1	2	2	3	3	3	2	2	2	2
T2SS-TAD (HGT-P)	2	4	4	1	1	2	3	2	2	2	1	1	1
T2SS-TAD (HGT-CS)	1	3	3	0	1	1	2	1	1	0	0	1	1
T3SS (total)	0	0	0	0	0	0	0	1	1	1	1	1	1
T3SS (HGT-P)	0	0	0	0	0	0	0	0	0	1	1	1	0
T3SS (HGT-CS)	0	0	0	0	0	0	0	0	0	1	1	0	0
T4SS (total)	0	0	0	1	0	1	0	1	1	0	0	0	0
T4SS (HGT-P)	0	0	0	1	0	1	0	1	1	0	0	0	0
T4SS (HGT-CS)	0	0	0	1	0	1	0	1	1	0	0	0	0
T6SS (total)	2	2	2	3	3	3	3	3	3	2	2	2	2
T6SS (HGT-P)	1	1	0	2	1	1	1	1	1	1	1	0	1
T6SS (HGT-CS)	1	1	1	0	0	0	0	1	1	0	0	0	0

#### Plasmid-mediated HGT

A total of 14 plasmids from 12 *V*. *coralliilyticus* strains originating from diverse hosts and geographic locations were compared. Ten plasmids exhibited >85% aa identity (Fig. 5, Table 3). Plasmids varied in size and structure, including prophage-derived insertions. A phage-derived insertion encoding two zona occludens toxin (*zot*) genes was identified on plasmids from *V. coralliilyticus* strains LU3DPEP4 (this study) and BAA-450 (Fig. 5). Across the set of ten plasmids, additional putative genes annotated as a cytotoxin MCF and a leucocidin domain-containing protein were detected (Fig. 5). Conjugative transfer system genes were also present. blast comparisons indicated that the 63 kb plasmid from *V. owensii* strain LU5DTRY1 shared 95% aa identity with an ~70 kb plasmid reported in pathogenic *V. parahaemolyticus* strains associated with AHPND in shrimp (Table 3). Comparative analyses showed that the LU5DTRY1 plasmid lacked *pirAB* toxin genes (Fig. 6). No additional annotated toxin genes were detected, although the plasmid encoded a predicted secreted trypsin-like peptidase belonging to an unclassified subgroup within the S1 peptidase family (MEROPS). Two additional plasmids from *V. tubiashii* strains TU1DMA1 (47 kbp) and TU1DMA2 (71 kbp) shared >90% aa identity to the 57-kbp plasmid from *V. tubiashii* strain ATCC 19109 (Table 3, Fig. S5). These plasmids did not contain any annotated virulence genes, though many encoded proteins were not functionally annotated (Fig. S5).

**Fig. 5.**
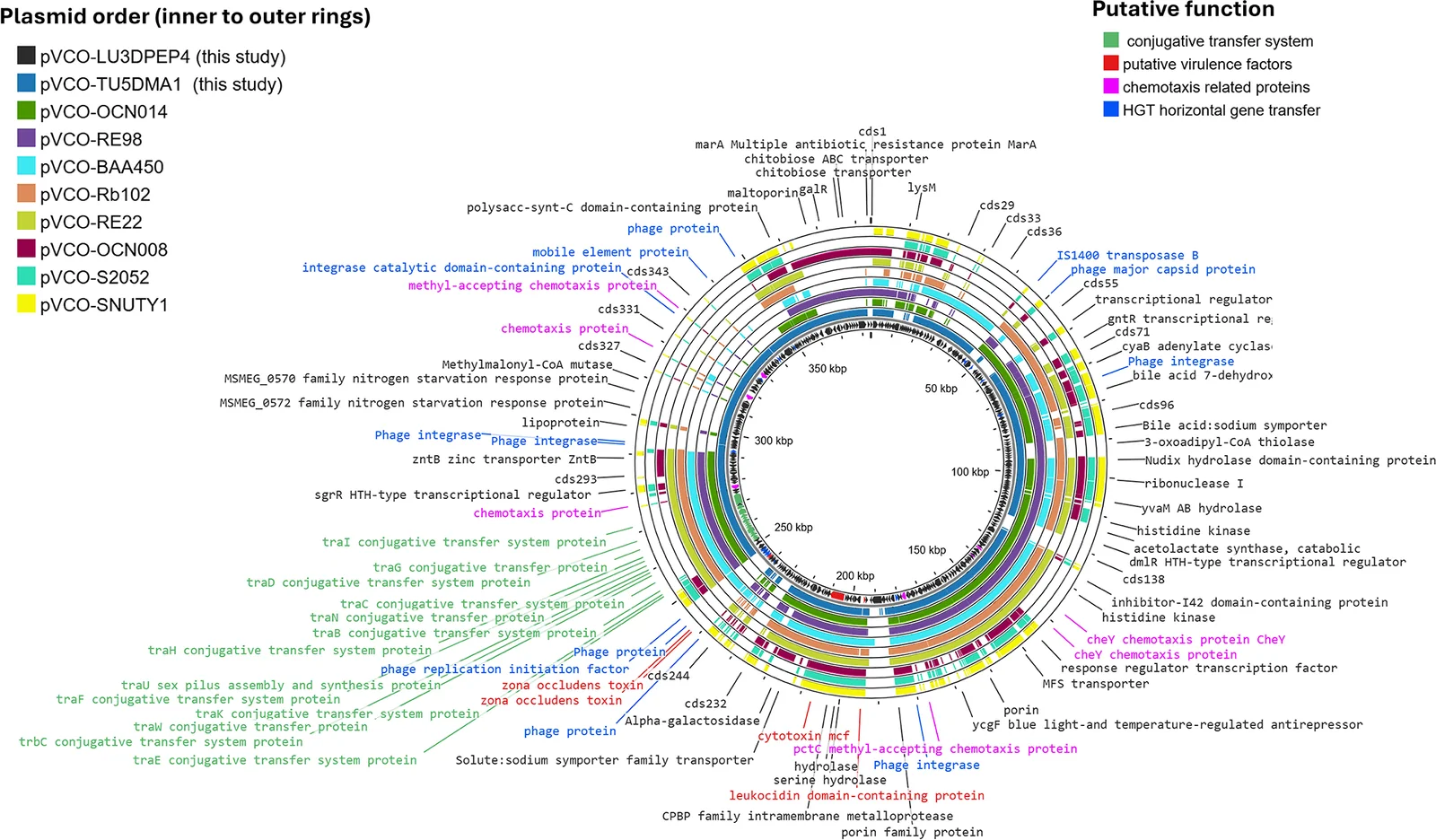
Comparative circular alignment of plasmids from ten *V. coralliilyticus* strains, including two plasmids sequenced in this study (pVCO-LU3DPEP4 and pVCO-TU5DMA1) and eight reference plasmids from the NCBI database (pVCO-OCN014, pVCO-RE98, pVCO-BAA450, pVCO-Rb102, pVCO-RE22, pVCO-OCN008, pVCO-S2052 and pVCO-SNUTY1; see Table 3 for strain information and accession numbers). Each concentric ring represents a single plasmid and is labelled with its corresponding plasmid name. Coloured features indicate functional gene categories, including conjugative transfer systems (green), putative virulence factors (red), chemotaxis-related proteins (pink) and HGT–associated genes (blue).

**Fig. 6.**
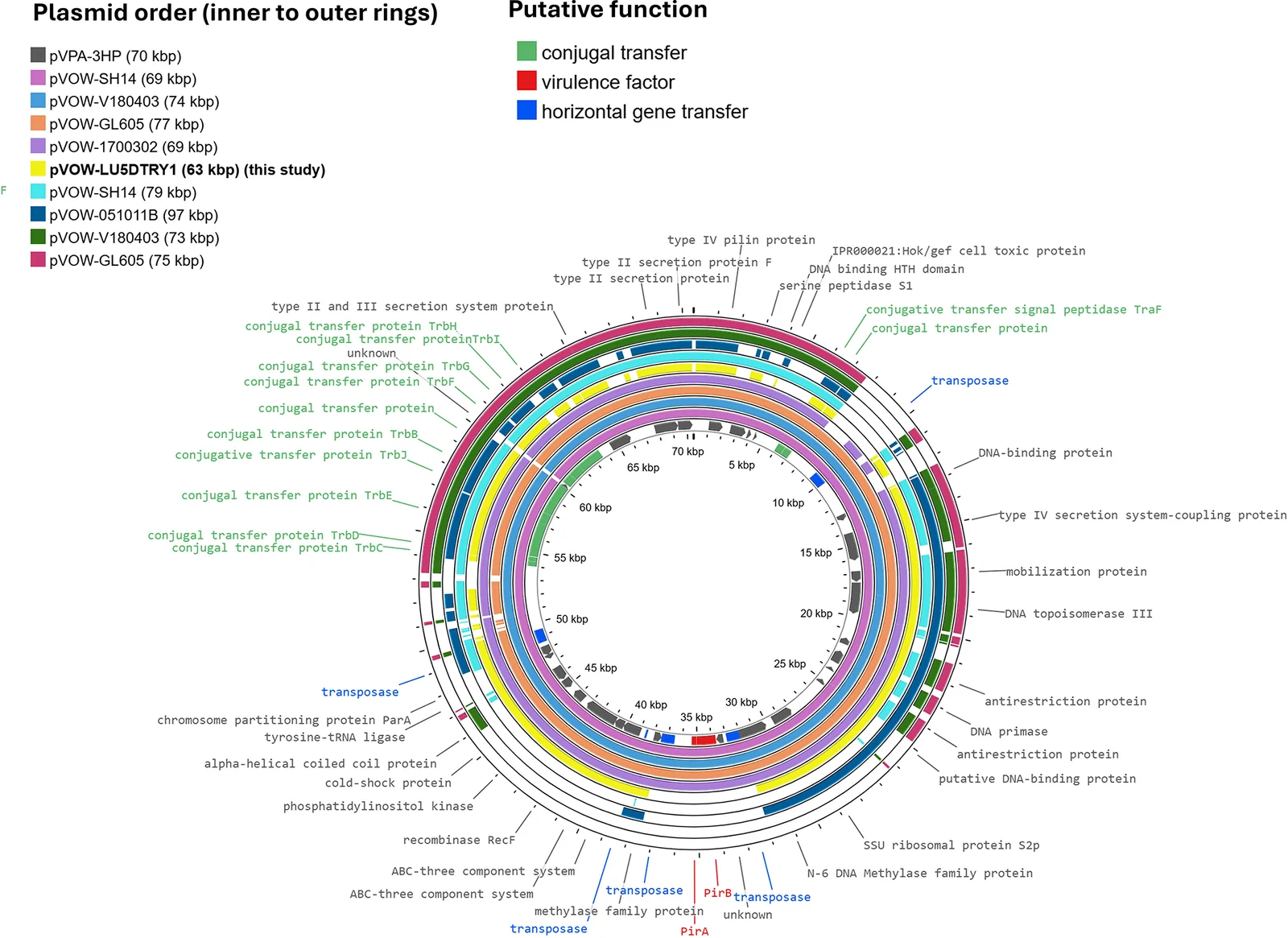
Comparative circular alignment of plasmids from nine strains of *V. owensii and* one strain of *V. parahaemolyticus*, including one plasmid from this study [pVOW-LU5DTRY1 (63 kbp)] and eight reference plasmids from the NCBI database [pVPA-3HP (70 kbp), pVOW-SH14 (69 kbp), pVOW-V180403 (74 kbp), pVOW-GL605 (77 kbp), pVOW-1700302 (69 kbp), pVOW-SH14 (79 kbp), pVOW-051011B (97 kbp), pVOW-V180403 (73 kbp) and pVOW-GL605 (75 kbp); see Table 3 for strain information and accession numbers]. Each concentric ring represents a single plasmid and is labelled with its corresponding plasmid name. Coloured features indicate functional gene categories, including conjugative transfer systems (green), putative virulence factors (red) and HGT–associated genes (blue).

#### Phage-mediated HGT

A total of 11 intact bacteriophages were identified across six *Vibrio* strains (Table 4, Figs 7 and S4). Nine phages were integrated into host chromosomes, one was located within a plasmid (pVCO-LU3DPEP4-384, Table 3) and one existed as a free circular phage (V.TUB.ph-73.8, Table 4). This episomal phage was initially annotated as a plasmid (pVTU-TU3DMA1-74) but was subsequently recognized as a phage maintained episomally during lysogeny [58]. Prophage annotation identified virulence-associated genes in three phage regions. Accessory cholera toxin (*ace*) and zona occludens toxin (*zot*) genes were detected in prophages V.COR.ph-12.3 and V.OWE.ph-120.6 (Fig. 7a, c). A prophage in *V. tetraodonis* (V.TET.ph-16.4) encoded a pectin lyase (Fig. 7b).

**Fig. 7.**
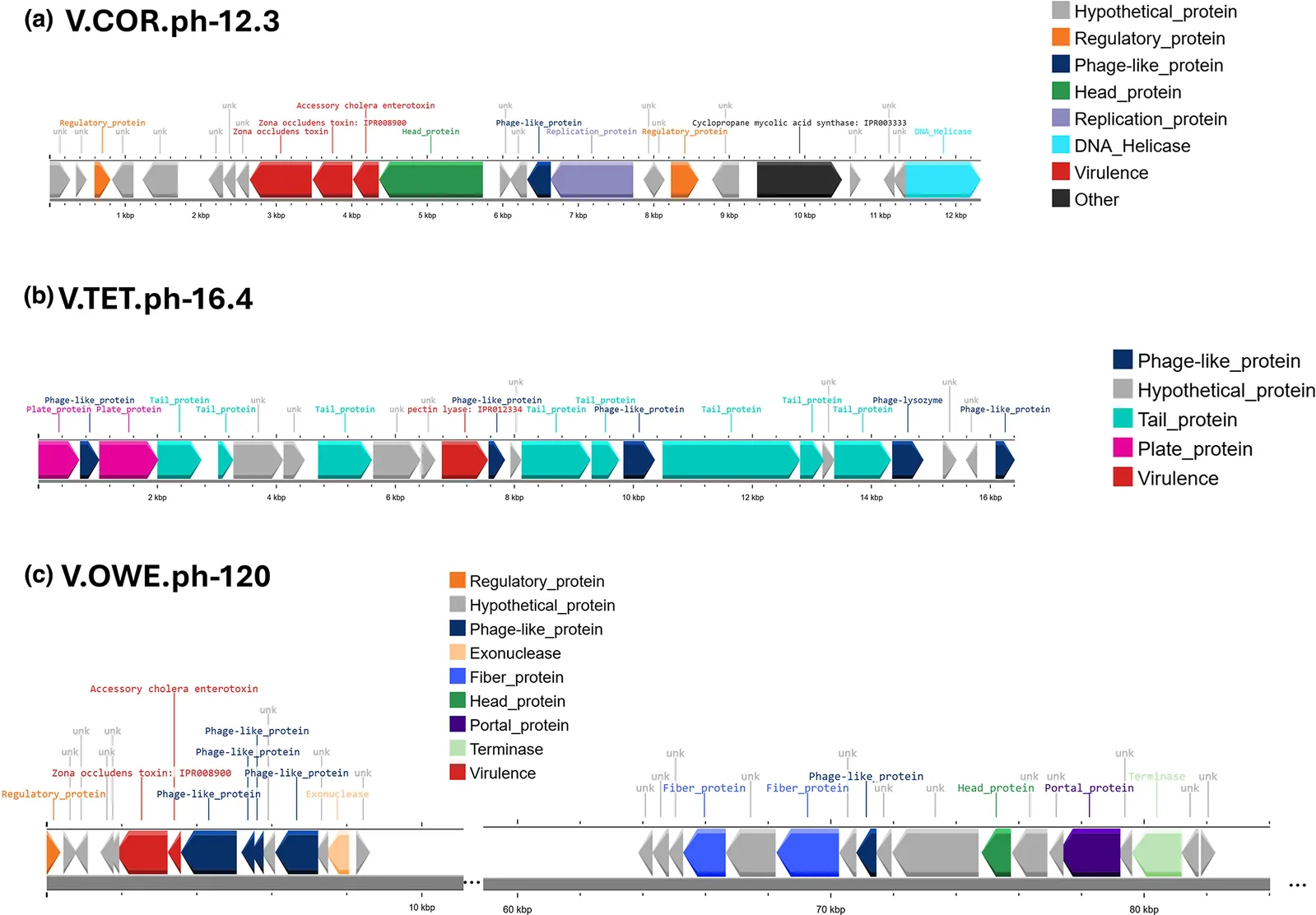
Virulence-associated prophage regions identified in coral-associated *Vibrio* genomes. Genomic organization of intact prophages carrying putative virulence-related genes in *Vibrio* strains isolated from *P. cylindrica*. (**a**) V.COR.ph-12.3 from *V. coralliilyticus* containing putative homologues of the accessory cholera enterotoxin (*ace*) and zonula occludens toxin (*zot*) genes. (**b**) V.TET.ph-16.4 from *V. tetraodonis* containing a homologue of a pectin lyase gene. (**c**) V.OWE.ph-120.6 from *V. owensii* containing *ace* and *zot* homologues. Arrows represent predicted ORFs and indicate gene orientation. Colours denote functional categories as indicated in the legend (e.g. phage structural proteins, replication proteins, regulatory proteins, virulence-associated genes and hypothetical proteins). Scale bars indicate genomic distance (kb). Prophage genome locations within host chromosomes or plasmids are provided in Table 4.

#### GIs and MGEs

Putative HGT regions were predicted using IslandViewer4 and AlienHunter (Table 5). IslandViewer4 identified between 2 and 89 GIs per chromosome, corresponding to 2–21% of chromosome length, whereas AlienHunter predicted 20–153 fragments per genome, covering 6–37% of each chromosome. Comparative genomic support provided an independent line of evidence for evaluating candidate HGT regions. Because both tools can generate false positives [59], only HGT regions containing virulence factors (Fig. 1) and/or secretion system genes (Fig. 2) were further evaluated using whole-chromosome alignments against reference genomes (Table 5; e.g. Fig. 8). We chose comparative genome alignment rather than consensus predictions between IslandViewer4 and AlienHunter because the two programs rely on different detection strategies and exhibit different sensitivities. T4SS gene clusters identified in *V. harveyi* genomes were located within GIs (Fig. 8). These islands contained at least two integrases and one relaxase, consistent with integrative and conjugative elements (ICEs) [60]. Although these ICEs lacked annotated toxin genes, they contained genes associated with adaptation (e.g. iron-scavenging and cold-shock proteins). In *V. owensii*, T4SS-bearing islands occurred at distinct genomic positions in TU8HTRY5 (large chromosome) and LU5DTRY1 (small chromosome), indicating variability in integration sites. T3SS gene clusters in the two *V. coralliilyticus* strains were located within regions homologous to a previously described pathogenicity island [35]. Comparative analysis indicated that all ten complete *V. coralliilyticus* genomes available in NCBI contained T3SS clusters (data not shown), whereas a closely related *Vibrio* sp. SCSIO 43001 (86% ANI to *V. coralliilyticus*) lacked the corresponding T3SS-containing island. Additional secretion system loci were also associated with predicted HGT regions (Table 5), including 15 of 36 T2SS-TAD clusters, 10 of 52 T1SS clusters and several T6SS clusters. In *V. tubiashii,* strains differed in the T6SS subtype present within HGT regions (Table 5).

**Fig. 8.**
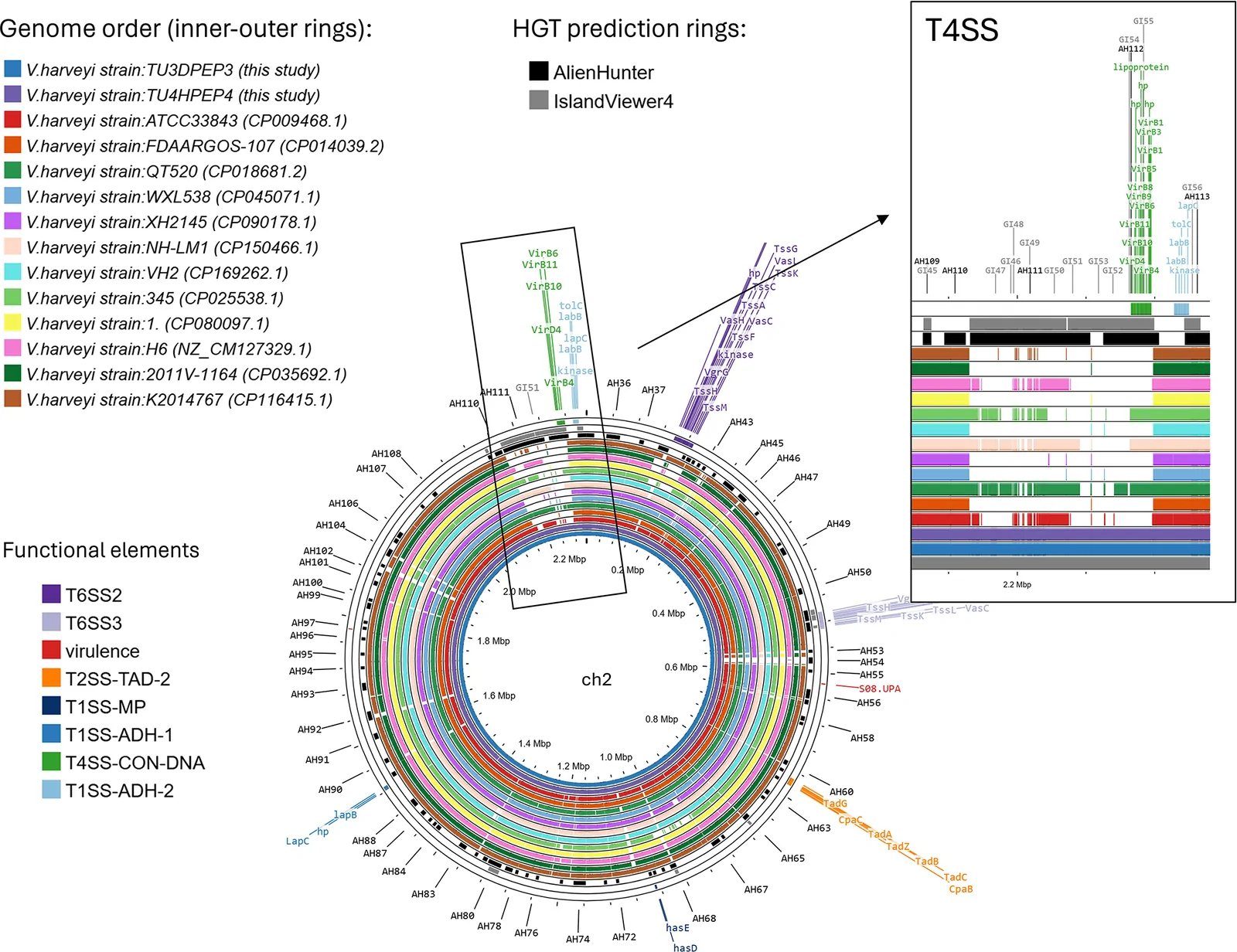
Comparative genomic context of a predicted HGT region carrying a type IV secretion system (T4SS) on chromosome 2 of *V. harveyi*. Circular genome comparison generated using Proksee showing chromosome 2 (ch2) of *V. harveyi* strains TU3DPEP3 and TU4HPEP4 (this study) as the innermost rings. The outer rings represent homologous regions from related *V. harveyi* reference genomes (NCBI accession number in parentheses). Coloured segments indicate regions of sequence similarity identified by blast-based alignment. Predicted HGT regions are shown as separate rings: IslandViewer4 (grey) and AlienHunter (black). Overlapping predictions between the two tools indicate regions with stronger computational support for horizontal acquisition. Functional elements detected within the focal region are highlighted and colour-coded, including T4SS components (green), T6SS clusters (purple), T2SS-TAD-2 (orange), T1SS subtypes (blue shades) and additional virulence-associated genes (red). Gene labels are shown for representative components within the enlarged inset panel. The insert displays a magnified view of the T4SS-containing GI, illustrating gene organization and correspondence with predicted HGT boundaries. Comparative absence or divergence of this region among reference genomes supports its potential horizontal acquisition.

## Discussion

### Distribution and genomic diversity of coral-associated *Vibrio*

In the current study, nearly half of the cultured bacterial isolates from *P. cylindrica* belonged to the genus *Vibrio*, underscoring the prominence of this group within the cultivable coral-associated microbiota. Among the five dominant *Vibrio* species isolated in this study, four *– V. owensii*, *V. tubiashii*, *V. coralliilyticus* and *V. harveyi –* have previously been identified as coral pathogens [10, 14, 16, 19, 20]. In contrast, the group classified as *V. tetraodonis/aquimaris* has not been associated with coral disease or pathogenicity in marine animals to date [61, 62]. However, *Vibrio* isolates were not restricted to diseased tissue; they were also obtained from visually healthy colonies, but at much smaller numbers. Although culture-based isolation does not directly correspond to *in situ* abundance and may be influenced by growth conditions, the recovery of established coral pathogens from both healthy and diseased tissues is consistent with observations that several *Vibrio* species can be present in asymptomatic coral hosts and behave as opportunistic pathogens under changing environmental conditions [63–65]. In many coral diseases, a single obligate pathogen is not consistently identified, and outbreaks have been associated with shifts in host physiology and microbiome structure, as well as environmental stressors such as elevated temperature [66–68]. Within this framework, the distribution of virulence-associated gene repertoires across multiple coral-associated *Vibrio* lineages suggests that pathogenic potential may be broadly distributed [64, 69]. The genomic architecture of the sequenced strains further highlights the diversity and plasticity of coral-associated *Vibrio*. Most genomes displayed the canonical two-chromosome structure typical of the genus, whereas one *V. tetraodonis* strain exhibited a single-chromosome configuration, potentially reflecting chromosomal fusion events similar to those described in *V. cholerae* [70, 71]. In addition, the presence of plasmids in multiple lineages indicates ongoing genetic exchange and the potential acquisition of accessory traits, as plasmid-mediated HGT has been shown to disseminate genes for adaptation, virulence and other functions across bacterial populations [72, 73]. Collectively, these findings suggest that coral-associated *Vibrio* populations comprise a genetically heterogeneous assemblage characterized by conserved genomic architecture, structural variation and mobile elements. This genomic diversity provides a foundation for exploring how virulence-associated genes, secretion systems and horizontally acquired elements may contribute to opportunistic pathogenicity and coral disease dynamics.

### Conserved and lineage-specific virulence architectures in coral-associated *Vibrio*

Comparative analysis of virulence-associated genes across thirteen *Vibrio* strains representing five species and both healthy and disease-associated coral tissues revealed a layered virulence architecture, comprising broadly conserved extracellular enzymes and more restricted toxin and regulatory modules, consistent with the modular organization of virulence determinants described in many bacterial pathogens [74–76]. Lineage-restricted genes such as *vvhA and hlyA* homologues [33, 35, 77], cholera accessory toxins and their associated regulators [31, 78, 79] as well as the *hcnABC* cluster [80–82] were confined to particular taxa, including *V. coralliilyticus* and *V. tetraodonis*. These genes represent candidate lineage-specific modules that may contribute to cytotoxicity or competitive interactions, although their functional roles remain to be experimentally validated [35, 74, 75]. Their restricted distribution suggests that while pathogenic potential may be broadly distributed within coral-associated *Vibrio* populations, certain lineages harbour additional virulence-associated modules that could influence host interactions or infection dynamics [64, 66, 69]. In contrast, extracellular proteases, including vibriolysins, collagenases, subtilisins [83–89] and the thermolabile haemolysin [90–92] were broadly distributed across taxa. Many of these enzymes have been implicated in virulence in other marine hosts. Yet, their universal or near-universal presence across strains from both healthy and diseased tissues suggests that they likely serve dual ecological roles. In addition to contributing to tissue degradation during infection, these enzymes may facilitate nutrient acquisition from organic substrates within the coral-associated microbiome.

### Core and modular secretion architectures in coral-associated *Vibrio*

The comparative genomic analysis across the 13 strains reveals a layered secretion system architecture across coral-associated *Vibrio* genomes, characterized by a conserved motility and adhesion core alongside lineage-specific injectosome and competitive modules, consistent with the modular organization of bacterial secretion systems described across Gram-negative pathogens [93, 94]. Flagellar systems and conserved T2SS components were present in all strains, consistent with a shared ecological foundation for motility, surface attachment and environmental persistence [24, 95, 96]. Variation in flagellar cluster organization and TAD copy number suggests differences in motility strategy and adhesion capacity among lineages [96, 97], but these systems were not restricted to isolates from diseased tissue. Greater lineage-level differentiation was observed in injectosome-type T3SS systems. T3SS3-like systems in *V. coralliilyticus* and *V. tetraodonis* and T3SS1-like systems in *V. harveyi* indicate diversification of host-targeting machinery [98–100]. However, T3SS was not exclusive to a single taxon, and predicted effectors were not confined to isolates from lesions, suggesting that host-targeting potential is modular rather than diagnostic of a specific pathogen [24, 93]. T4SS systems were frequently associated with plasmids, highlighting their role in HGT and genomic plasticity [101–103]. Their distribution across multiple species underscores the importance of mobile elements in shaping secretion system diversity and facilitating gene exchange [104]. T6SS exhibited both conserved and lineage-specific subtypes. The universal presence of TssM–VgrG suggests a shared competitive or host-interaction function [105, 106], while variation in additional subtypes indicates diversification of interbacterial and potentially host-targeting capacities [107]. Although *V. coralliilyticus* strains carried a broader repertoire of predicted anti-eukaryotic effectors [108], homologues were also present in other *Vibrio* isolates, reinforcing the distributed nature of potential virulence mechanisms. Overall, the diversity of secretion systems in coral-associated *Vibrio* reflects a modular, evolutionarily dynamic architecture [93, 94]. A conserved colonization toolkit is shared across taxa, while variable injectosome and competitive systems may modulate ecological interactions and pathogenic potential [24, 105]. Importantly, no single secretion system uniquely defined isolates recovered from diseased tissues, supporting the hypothesis that virulence-associated traits are distributed across multiple lineages and shaped by genomic plasticity rather than by a discrete obligate pathogen [64, 66, 69].

### HGT as a driver of virulence modularity and genomic plasticity in coral-associated *Vibrio*

Across these coral-associated *Vibrio* genomes, horizontally acquired elements – including plasmids, prophages/episomal phages and GIs – represent major sources of accessory genetic variation, consistent with the recognized role of HGT in shaping *Vibrio* ecology and evolution [109–111]. The plasmid comparisons indicate that plasmids can be highly conserved within lineages (e.g. across multiple *V. coralliilyticus* strains) yet structurally dynamic due to insertions such as prophage-derived regions [58, 112]. Notably, only a subset of plasmids carried identifiable virulence-associated genes (e.g. *zot* insertions), suggesting that many plasmids may function primarily as vehicles for gene exchange and adaptation rather than direct determinants of pathogenicity [103, 113]. However, plasmid-mediated acquisition of virulence determinants is well documented in *Vibrio*, including toxin-bearing plasmids associated with AHPND in shrimp [38, 114–116], illustrating the potential for rapid phenotypic shifts following plasmid transfer [60]. Phage-associated loci contributed additional virulence-related traits, including *ace* and *zot* genes and a pectin lyase encoded within prophage regions. Prophages are well known to mediate the transfer of virulence genes and genomic innovation in *Vibrio* spp. and other pathogens [109, 111]. The identification of a prophage-associated pectin lyase is consistent with documented lateral acquisition of polysaccharide-degrading pathways in marine *Gammaproteobacteria* [117] and with the established virulence of pectic enzymes in plant-associated bacteria [118], suggesting potential ecological or host-associated functionality in marine systems. Beyond direct gene carriage, the presence of multiple intact prophages across strains highlights the potential for phage-mediated genome remodelling and gene movement within coral-associated microbial communities [113, 119, 120]. Notably, plasmids and prophages were detected exclusively in strains isolated from diseased coral tissues, whereas GIs were identified in both healthy- and disease-associated isolates. This observation should be interpreted cautiously because only 4 of the 13 sequenced genomes were recovered from visually healthy coral tissue, whereas 9 originated from diseased tissue. Consequently, the apparent restriction of plasmids and prophages to disease-associated isolates may reflect sampling imbalance rather than a true biological association. However, the consistent association of plasmids and phage-related elements with disease-derived strains, together with their enrichment in virulence-associated genes (e.g. *zot* and *ace*) and conjugative systems, is consistent with the established role of these elements in mediating pathogenicity and rapid adaptation in *Vibrio* [38, 109–111]. These observations are consistent with the hypothesis that MGEs may contribute to enhanced virulence potential in strains associated with diseased coral tissues. However, broader sampling will be required to determine whether the observed pattern reflects a consistent biological difference.

GIs served as key integration platforms for secretion systems and other adaptive traits. The placement of T4SS clusters within ICE-like islands and the localization of T3SS clusters within pathogenicity-island homologues support the view that host interaction machinery in *Vibrio* can be gained, lost or rearranged through horizontal transfer [28, 121, 122]. The variability in island location and content among closely related strains further indicates ongoing genomic flux, a hallmark of *Vibrio* eco-evolutionary dynamics [110]. While bioinformatic prediction of GIs can vary across detection tools [59], the repeated localization of secretion systems within predicted HGT regions is consistent with horizontal acquisition. Taken together, these findings support the hypothesis that *Vibrio* virulence potential is not encoded solely within the conserved core genome but is shaped by a modular accessory gene pool redistributed through HGT [109–111]. Such genomic plasticity is consistent with an evolutionary mechanism for rapid shifts in host interaction strategies and may contribute to opportunistic disease dynamics in coral-associated *Vibrio* populations [24, 35].

Because genome sequencing was performed on a small, representative subset of isolates selected to maximize species, geographic and tissue diversity rather than to generate statistically balanced groups, formal statistical comparisons of virulence-associated gene abundance, secretion system composition and MGEs among species or health states were not appropriate. Consequently, the comparative genomic analyses presented here are intended to identify patterns and generate hypotheses regarding genomic features potentially associated with coral host interactions, rather than to establish statistically supported differences among taxa or health states. Expanded genome sampling will be required to test these hypotheses quantitatively.

Although comparative genomics provides a powerful framework for identifying candidate virulence-associated genes and reconstructing patterns of genome evolution, it cannot establish gene expression, protein activity or pathogenic function. Consequently, the genomic features identified in this study should be viewed as hypotheses regarding mechanisms of coral-associated virulence that require future validation through transcriptomic analyses, targeted mutagenesis and infection experiments. Integrating comparative genomics with functional approaches will be essential for determining how these genomic differences influence host interactions and disease progression in *P. cylindrica*.

## Conclusions

Comparative genomic analysis of five *Vibrio* species and 13 strains isolated from healthy and WS–affected *P. cylindrica* revealed a modular virulence landscape distributed across multiple lineages. All strains possessed conserved extracellular proteases, including vibriolysins, collagenases and subtilases, consistent with a shared ecological toolkit for protein degradation and host association. In contrast, cytolysins (e.g. *vvhA* and *hlyA* homologues) and phage-associated cholera-related toxins were restricted to particular taxa, reflecting lineage-specific acquisition of accessory virulence modules. Among the genomes examined, *V. coralliilyticus* contained the largest repertoire of identified virulence-associated gene homologues, followed by select *V. tetraodonis* and *V. owensii* strains, whereas *V. harveyi* and *V. tubiashii* harboured fewer identifiable virulence homologues. Rather than defining a single obligate pathogen, these patterns support a continuum of pathogenic potential in coral-associated *Vibrio*, in which conserved ecological functions are complemented by lineage-specific enhancements that may intensify infection under permissive environmental or host conditions. Substantial variation in secretion systems and MGEs further highlights the genomic plasticity of coral-associated *Vibrio*. Core colonization machinery, including flagellar systems and conserved T2SS components, was shared across taxa, whereas injectosome-type T3SS and conjugative T4SS systems exhibited lineage-level variability consistent with horizontal acquisition. The diversity of T6SS subtypes suggests both conserved competitive functions and diversification of interbacterial or host-targeting capacities.

Prophages and plasmids contributed additional layers of genomic flexibility, reinforcing the potential role of HGT in redistributing virulence-associated modules and reshaping host-interaction potential. Plasmids and prophages were detected exclusively in strains isolated from diseased coral tissues in this dataset, whereas GIs were present in both healthy- and disease-associated isolates. As discussed above, this observation should be interpreted cautiously because most sequenced genomes originate from diseased corals. Consequently, the apparent association of plasmids and prophages with disease-associated isolates may reflect sampling imbalance rather than a true biological difference. Expanded genomic sampling of healthy coral-associated *Vibrio* will be necessary to evaluate this pattern further.

Taken together, our comparative genomic analyses support the hypothesis that virulence-associated traits in coral-associated *Vibrio* are organized as a conserved ecological core complemented by a flexible accessory gene pool shaped, in part, by HGT. Likewise, the widespread distribution of virulence-associated gene homologues across multiple *Vibrio* species suggests that pathogenic potential may not be restricted to a single lineage but instead may be broadly distributed among opportunistic coral-associated *Vibrio*. Because these conclusions are based on comparative genomic analyses, future studies integrating functional assays, gene expression analyses and infection experiments will be required to determine the biological significance of these genomic features and their contribution to coral disease.

## Supplementary material

10.1099/mgen.0.001862 Supplementary Material 1.

10.1099/mgen.0.001862 Supplementary Material 2.

## Abbreviations

AHPND: acute hepatopancreatic necrosis disease
ANI: average nucleotide identity
GIs: genomic islands
HGT: horizontal gene transfer
ICE: integrative and conjugative element
MA: marine agar
MGEs: mobile genetic elements
PEP: peptone seawater media
RAST: Rapid Annotation using Subsystem Technology
TCBS: thiosulphate citrate bile salts agar
TRY: tryptone seawater media
WS: white syndrome
