# Supplementary material for: Comparative genomics reveals modular virulence repertoires and extensive horizontal gene transfer in Vibrio species associated with white syndrome of Porites cylindrica
Source: Microb Genom. 2026 Oct 23;12(10):001862. doi: 10.1099/mgen.0.001862 (PMC13694112; doi:10.1099/mgen.0.001862)
Supplement: Supplementary file 1 [file mgen-12-01862-s002.pdf]

Supplementary Table S1: Number and taxonomic classification of bacterial isolates obtained from healthy and diseased tissue of *Porites cylindrica* samples from two reef locations

		Luminao		Tumon Bay	
		Healthy	Diseased	Healthy	Diseased
Gammaproteobacteria	Vibrionaceae	4	21	8	25
Alphaproteobacteria	Rhodobacteraceae	15	15	0	0
Alphaproteobacteria	Kiloniellaceae	0	9	0	0
Alphaproteobacteria	Eilatimonas	2	0	0	0
Alphaproteobacteria	Rhizobiaceae	1	0	0	0
Alphaproteobacteria	Stappiaceae	2	0	5	0
Bacteroidia	Cyclobacteriaceae	0	1	0	0
Bacteroidia	Flavobacteriaceae	3	0	2	0
Actinobacteria	Micrococcales	0	2	0	0

Supplementary Table S2: Rationale for selecting representative *Vibrio* isolates for whole-genome sequencing. Representative isolates were selected from the five most frequently recovered *Vibrio* species to maximize representation of reef sites (Tumon and Luminao) and coral health states (healthy and diseased), with random selection applied when multiple isolates met these criteria.

Species	Total isolates	Genomes sequenced	Selection rationale
V. owensii	11	4	Isolates randomly distributed btw healthy and diseased sites, one from each tissue type and one from each site selected at random
V. tubiashii	11	3	Mostly isolated from Tumon diseased corals (10) – 3 representatives selected at random
V. tetraodonis	11	2	Most isolates (10) from diseased tissue at Luminao – 2 representatives selected at random
V. coralliilyticus	10	2	Most isolates from diseased tissue (10) included representatives from both sites, Tumon and Luminao, at random
V. harveyi	5	3	Isolated only from coral tissue in Tumon, 2 representatives – one from diseased and one from healthy at random

Supplementary Table S3 Average nucleotide identity (ANI) values between the 13 genomes sequenced in this study and the type strain genomes of their closest phylogenetic relatives identified by maximum-likelihood analysis.

Reference species	Reference NCBI assembly accession	Isolate name -this study	ANI (%)	Reference genome fragments	Query genome fragments
Vibrio owensii	GCF_002021755.1	LU5DTRY1	96.9907	1963	1846
Vibrio owensii	GCF_002021755.1	TU3DMA2	97.0455	1963	1848
Vibrio owensii	GCF_002021755.1	LU8HTRY1	97.0439	1963	1855
Vibrio owensii	GCF_002021755.1	TU8HTRY5	97.0232	1963	1843
Vibrio harveyi	GCF_030060435.1	TU3DPEP3	98.5397	1956	1793
Vibrio harveyi	GCF_030060435.1	TU4HPEP4	98.4822	1956	1801
Vibrio coralliilyticus	GCF_029541605.1	LU3DPEP4	97.2075	1918	1768
Vibrio coralliilyticus	GCF_029541605.1	TU5DMA1	97.2853	1918	1777
Vibrio tubiashii	GCF_000772105.1	TU1DMA1	95.6602	1843	1500
Vibrio tubiashii	GCF_000772105.1	TU3DMA1	95.7478	1843	1493
Vibrio tubiashii	GCF_000772105.1	TU1DMA2	95.6083	1843	1498
Vibrio aquimaris	GCF_009363415.1	LU3DMA2	94.6136	1499	1291
Vibrio aquimaris	GCF_009363415.1	LU5DMA4	94.8863	1499	1345
Vibrio tetraodonis	GCF_003350295.1	LU3DMA2	95.1874	1435	1279
Vibrio tetraodonis	GCF_003350295.1	LU5DMA4	95.4677	1435	1299

Supplementary Figure S1. Maximum-likelihood phylogenomic tree based on the GTDB-Tk bac120 marker set from 189 taxa, including 175 publicly available *Vibrio* reference genomes, 13 isolates sequenced in this study, and one outgroup taxon (*Aliivibrio fischeri*). The phylogeny was reconstructed from the concatenated amino acid alignment of 120 conserved bacterial marker genes using RAxML v8.2.12 under the PROTGAMMAILGF model. Branch support was assessed using 100 rapid bootstrap replicates, with bootstrap values ≥80% shown at the nodes. Isolates sequenced in this study are highlighted in blue. The tree was rooted using *A. fischeri* as the designated outgroup. The scale bar indicates amino acid substitutions per site.

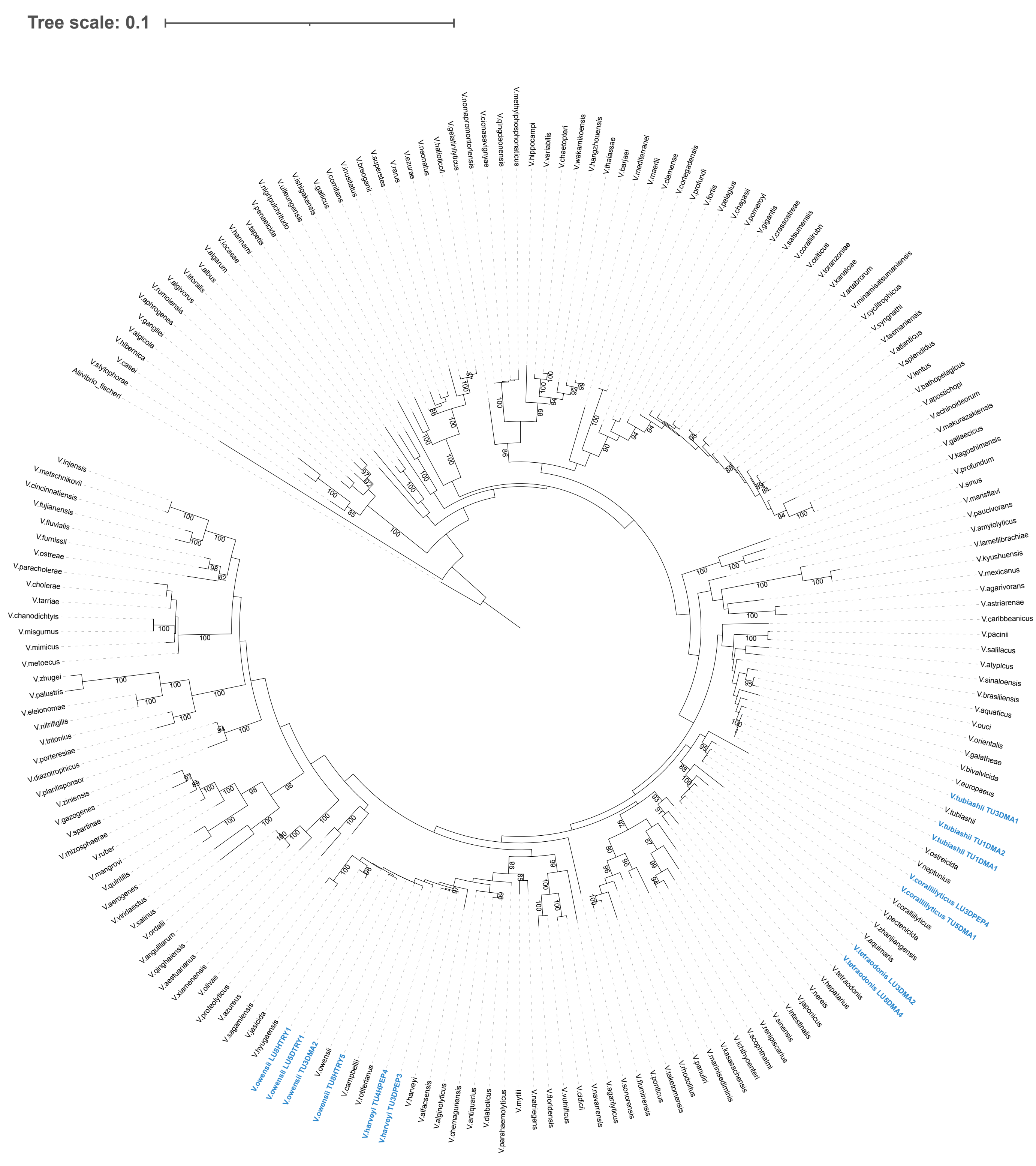
Tree scale: 0.1
Aliivibrio_fischeri
V.stylophorae
V.casei
V.hibernica
V.algicola
V.ganglilei
V.aphrogenes
V.rumoiensis
V.algivorus
V.litoralis
V.albus
V.algarum
V.iocasae
V.hannami
V.tapetis
V.penaeicida
V.nigripulchritudo
V.ulleungensis
V.ishigakensis
V.gallicus
V.comitans
V.inusitatus
V.breoganii
V.superstes
V.rarus
V.ezurae
V.neonatus
V.halioticoli
V.gelatinilyticus
V.nomapromontoriensis
V.cionasavignyae
V.qingdaonensis
V.methylphosphonaticus
V.hippocampi
V.variabilis
V.chaetopteri
V.wakamikoensis
V.hangzhouensis
V.thalassae
V.barjaei
V.mediterranei
V.maerlii
V.clamense
V.cortegadensis
V.profundi
V.fortis
V.pelagius
V.chagasii
V.pomeroyi
V.gigantis
V.crassostreae
V.satsumensis
V.coralliirubri
V.celticus
V.toranzoniae
V.kanaloae
V.artabrorum
V.minamisatsumaniensis
V.cyclitrophicus
V.syngnathi
V.tasmaniensis
V.atlanticus
V.splendidus
V.lentus
V.bathopelagicus
V.apostichopi
V.echinoideorum
V.makurazakiensis
V.gallaecicus
V.kagoshimensis
V.profundum
V.sinus
V.marisflavi
V.paucivorans
V.amylolyticus
V.lamellibrachiae
V.kyushuensis
V.mexicanus
V.agarivorans
V.astriarenae
V.caribbeanicus
V.pacinii
V.salilacus
V.atypicus
V.sinaloensis
V.brasiliensis
V.aquaticus
V.ouci
V.orientalis
V.galatheae
V.bivalvicida
V.europaeus
V.tubiashii TU3DMA1
V.tubiashii
V.tubiashii TU1DMA2
V.tubiashii TU1DMA1
V.ostreicida
V.neptunius
V.coralliilyticus LU3DPEP4
V.coralliilyticus TU5DMA1
V.coralliilyticus
V.pectenicida
V.zhanjiangensis
V.aquimaris
V.tetraodonis LU3DMA2
V.tetraodonis LU5DMA4
V.tetraodonis
V.hepatarius
V.nereis
V.japonicus
V.intestinalis
V.sinensis
V.renipiscarius
V.scophthalmi
V.ichthyoenteri
V.kasasachensis
V.marisedimins
V.panuliri
V.modiolus
V.taketomensis
V.ponticus
V.fluminensis
V.sonorensis
V.agarilyticus
V.navarrensis
V.cidicii
V.vulnificus
V.floridensis
V.natriegens
V.mytili
V.parahaemolyticus
V.diabolicus
V.antiquarius
V.chemaguriensis
V.alginolyticus
V.alfacsensis
V.harveyi
V.harveyi TU3DPEP3
V.harveyi TU4HPEP4
V.rotiferianus
V.campbellii
V.owensii TU8HTRY5
V.owensii
V.owensii TU3DMA2
V.owensii LU5DTRY1
V.owensii LU8HTRY1
V.hyugaensis
V.jasicida
V.sagamiensis
V.azureus
V.proteolyticus
V.olivae
V.xiamenensis
V.aestuarianus
V.qinghaiensis
V.anguillarum
V.ordalii
V.salinus
V.viridaestus
V.aerogenes
V.quintilis
V.mangrovi
V.ruber
V.rhizosphaerae
V.spartinae
V.gazogenes
V.ziniensis
V.plantisponsor
V.diazotrophicus
V.porteresiae
V.tritonius
V.nitrifigilis
V.eleionomae
V.palustris
V.zhugei
V.metoecus
V.mimicus
V.misgurnus
V.chanodichtyis
V.tarriae
V.cholerae
V.paracholerae
V.ostreae
V.furnissii
V.fluvialis
V.fujianensis
V.cincinnatiensis
V.metschnikovii
V.injensis

Supplementary Figure S2. Comparative BLAST alignment of *Vibrio coralliilyticus* strains to the CP-1 pathogenicity island. BLAST-based genomic comparison of *Vibrio coralliilyticus* strains LU3DPEP4 and TU5DMA1 (this study) against the CP-1 pathogenicity island from *V. coralliilyticus* strain BAA-450 (Kimes et al., 2012). The top panel shows gene organization of the CP-1 island (reference), with genes color-coded by functional category: T3SS components (cyan), putative virulence factors (magenta), other annotated functions (brown), and hypothetical proteins (gray). Horizontal alignment tracks below indicate regions of nucleotide similarity between the reference CP-1 island (BAA-450) and the genomes of LU3DPEP4 and TU5DMA1. Colored bars represent BLAST matches meeting the applied similarity thresholds, illustrating conservation and structural variation across strains. Conserved T3SS structural genes (e.g., yscC, yscN, yscT, yscU) and associated effector and regulatory elements are present in both strains, although differences in gene content and synteny are evident, suggesting partial conservation of the CP-1 island among *V. coralliilyticus* isolates.

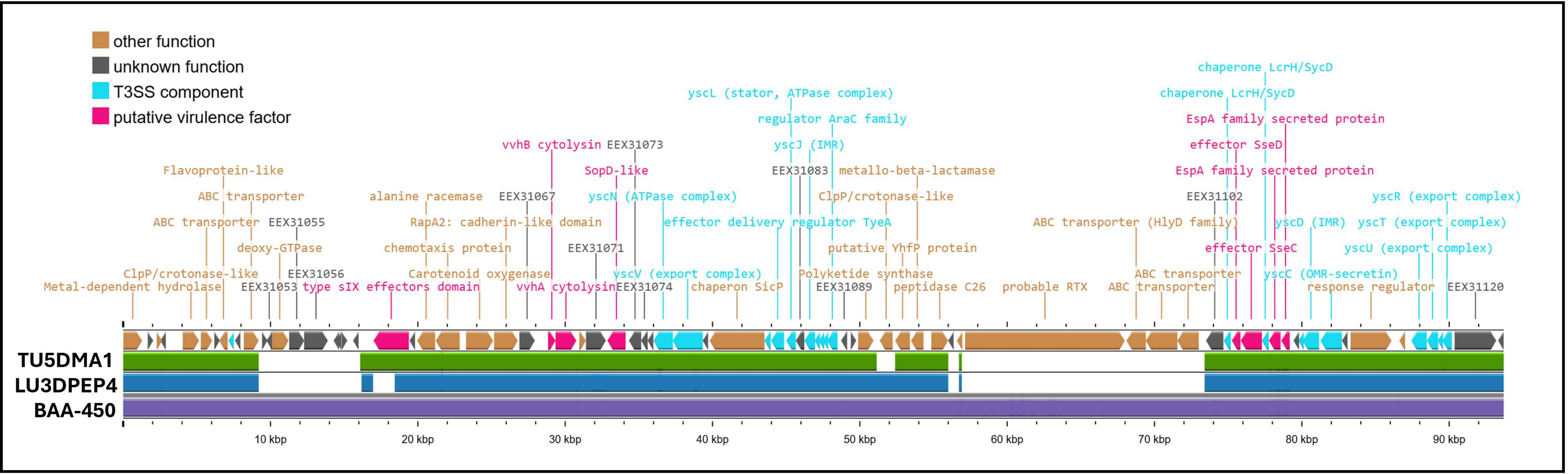
other function
unknown function
T3SS component
putative virulence factor
chaperone LcrH/SycD
yscL (stator, ATPase complex)
chaperone LcrH/SycD
regulator AraC family
EspA family secreted protein
vvhB cytolysin
EEX31073
yscJ (IMR)
effector SseD
Flavoprotein-like
SopD-like
EEX31083
metallo-beta-lactamase
EspA family secreted protein
ABC transporter
alanine racemase
EEX31067
yscN (ATPase complex)
ClpP/crotonase-like
EEX31102
yscR (export complex)
ABC transporter
EEX31055
RapA2: cadherin-like domain
effector delivery regulator TyeA
ABC transporter (HlyD family)
yscD (IMR)
yscT (export complex)
deoxy-GTPase
chemotaxis protein
EEX31071
putative YhfP protein
effector SseC
yscU (export complex)
ClpP/crotonase-like
EEX31056
Carotenoid oxygenase
yscV (export complex)
Polyketide synthase
ABC transporter
yscC (OMR-secretin)
Metal-dependent hydrolase
EEX31053
type sIX effectors domain
vvhA cytolysin
EEX31074
chaperon SicP
EEX31089
peptidase C26
probable RTX
ABC transporter
response regulator
EEX31120
TU5DMA1
LU3DPEP4
BAA-450
10 kbp
20 kbp
30 kbp
40 kbp
50 kbp
60 kbp
70 kbp
80 kbp
90 kbp

Supplementary Figure S3. Amino acid alignments of selected virulence-associated proteins identified in *Vibrio* isolates from this study. Multiple sequence alignments comparing representative virulence-associated proteins identified in this study with reference toxins from established pathogens. Percent amino acid identity relative to reference sequences is indicated for each homolog. (A) HlyA homologs aligned to *Vibrio cholerae* hemolysin (WP_001125271). **(**B) *VvhA* homologs aligned to *Vibrio vulnificus* cytolysin (WP_011081361). (C) Ace (accessory cholera enterotoxin) homologs aligned to *V. cholerae* Ace (KWW43308). (D) *Zot*, zonula occludens toxin homologs aligned to *V. cholerae* (WP_275082003, WP_316245170). (E) *HcnABC* hydrogen cyanide synthase subunits aligned to reference sequences from *Pseudomonas aeruginosa* PAO1 (AAF21028, AAF21029A, AF21030). Conserved domains annotated in reference proteins are shown above each alignment (e.g., leukocidin family pore-forming toxin domain, beta-trefoil ricin-like hemolysin domain). Green bars indicate conserved regions, and black/gray shading represents amino acid conservation across aligned sequences.

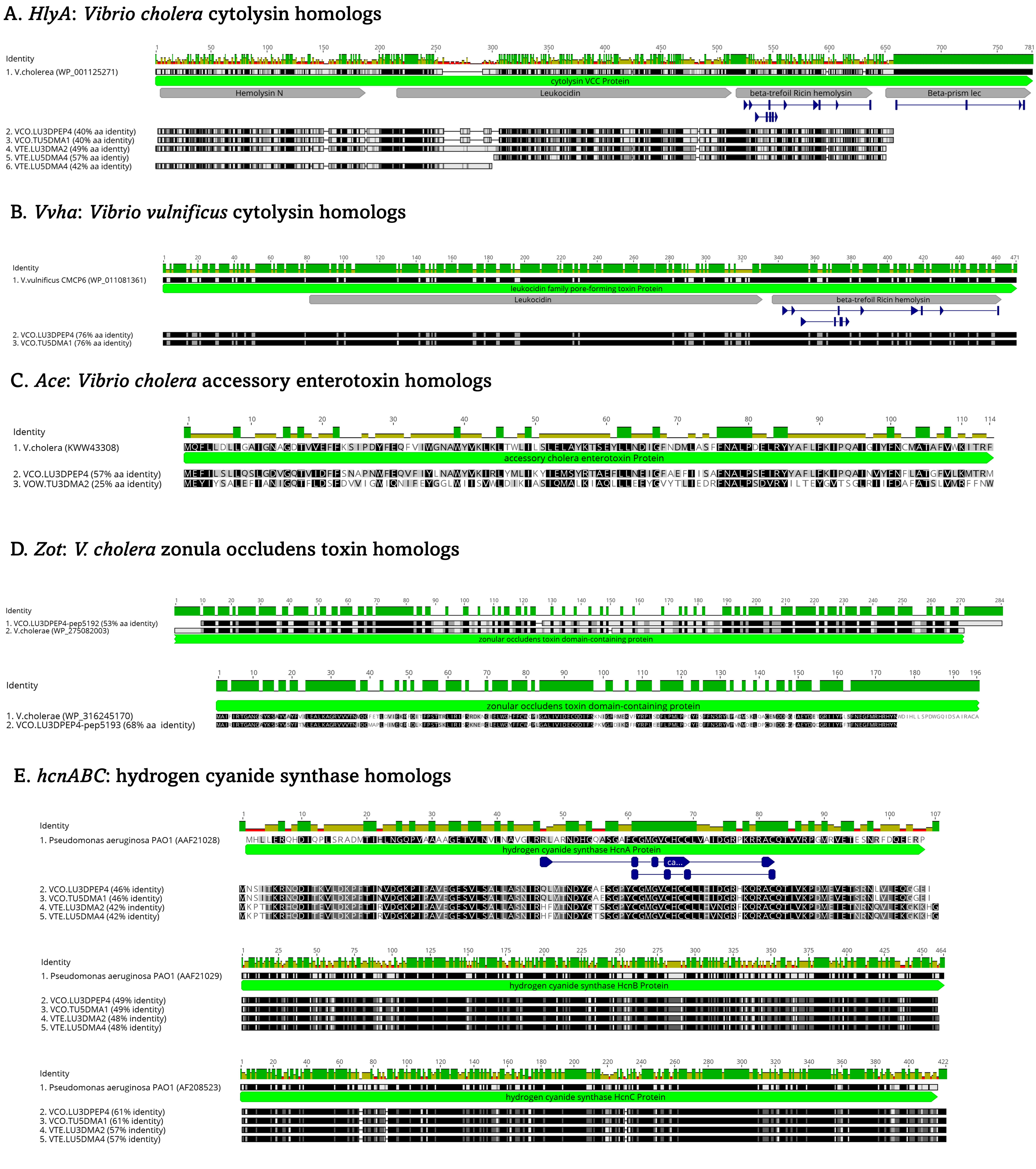
A. HlyA: Vibrio cholera cytolysin homologs
Identity
1. V.cholerea (WP_001125271)
cytolysin VCC Protein
Hemolysin N
Leukocidin
beta-trefoil Ricin hemolysin
Beta-prism lec
2. VCO.LU3DPEP4 (40% aa identity)
3. VCO.TU5DMA1 (40% aa identity)
4. VTE.LU3DMA2 (49% aa identy)
5. VTE.LU5DMA4 (57% aa identy)
6. VTE.LU5DMA4 (42% aa identity)
B. Vvha: Vibrio vulnificus cytolysin homologs
Identity
1. V.vulnificus CMCP6 (WP_011081361)
leukocidin family pore-forming toxin Protein
Leukocidin
beta-trefoil Ricin hemolysin
2. VCO.LU3DPEP4 (76% aa identity)
3. VCO.TU5DMA1 (76% aa identity)
C. Ace: Vibrio cholera accessory enterotoxin homologs
Identity
1. V.cholera (KWW43308)
accessory cholera enterotoxin Protein
2. VCO.LU3DPEP4 (57% aa identity)
3. VOW.TU3DMA2 (25% aa identity)
D. Zot: V. cholera zonula occludens toxin homologs
Identity
1. VCO.LU3DPEP4-pep5192 (53% aa identity)
2. V.cholerae (WP_275082003)
zonular occludens toxin domain-containing protein
Identity
zonular occludens toxin domain-containing protein
1. V.cholerae (WP_316245170)
2. VCO.LU3DPEP4-pep5193 (68% aa identity)
E. hcnABC: hydrogen cyanide synthase homologs
Identity
1. Pseudomonas aeruginosa PAO1 (AAF21028)
hydrogen cyanide synthase HcnA Protein
2. VCO.LU3DPEP4 (46% identity)
3. VCO.TU5DMA1 (46% identity)
4. VTE.LU3DMA2 (42% indentity)
5. VTE.LU5DMA4 (42% identity)
Identity
1. Pseudomonas aeruginosa PAO1 (AAF21029)
hydrogen cyanide synthase HcnB Protein
2. VCO.LU3DPEP4 (49% identity)
3. VCO.TU5DMA1 (49% identity)
4. VTE.LU3DMA2 (48% indentity)
5. VTE.LU5DMA4 (48% identity)
Identity
1. Pseudomonas aeruginosa PAO1 (AF208523)
hydrogen cyanide synthase HcnC Protein
2. VCO.LU3DPEP4 (61% identity)
3. VCO.TU5DMA1 (61% identity)
4. VTE.LU3DMA2 (57% indentity)
5. VTE.LU5DMA4 (57% identity)

Supplementary Figure S4. Genomic organization of intact prophages identified in *Vibrio* genomes from this study. Gene maps of intact prophages detected using PHASTER in thirteen *Vibrio* strains isolated from *Porites cylindrica*. Prophage names correspond to those listed in Table 4. Arrows represent predicted open reading frames and indicate transcriptional orientation. Genes are color-coded by functional category (e.g., head proteins, tail proteins, portal proteins, terminase, integrase, replication proteins, regulatory proteins, transposases, hypothetical proteins). Scale bars indicate genomic distance (kb). These prophages vary in size and gene composition but share conserved structural modules typical of temperate bacteriophages. While most prophages encode structural and replication functions, only a subset carry identifiable virulence-associated genes (see Figure 7), highlighting functional heterogeneity among prophage elements.

V.TET.ph-16.5

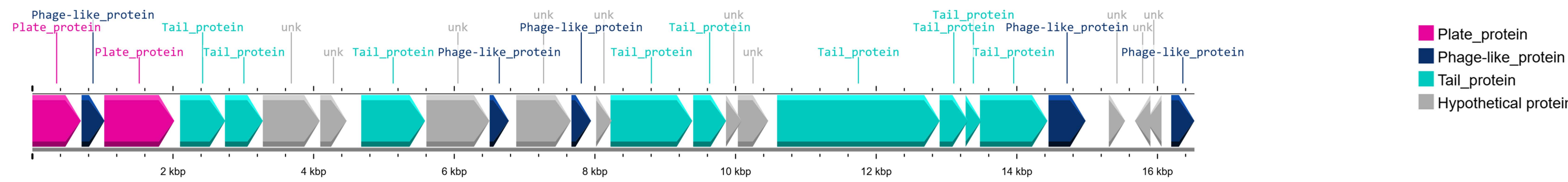

V.TET.ph-35.0

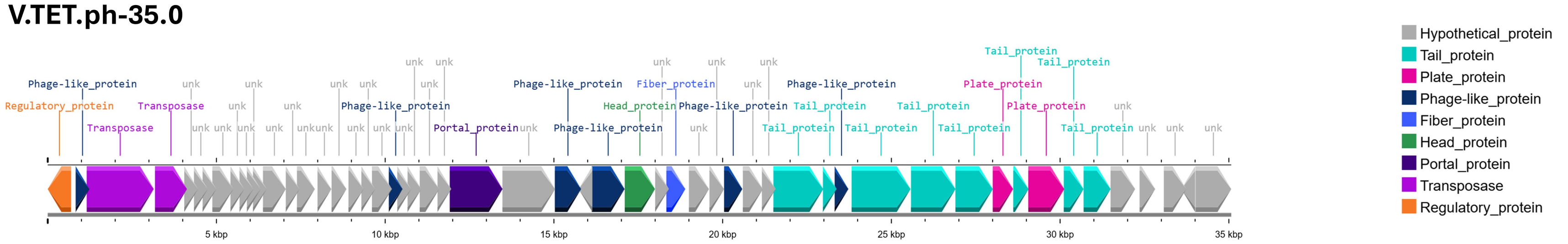

V.TUB.ph-27.2

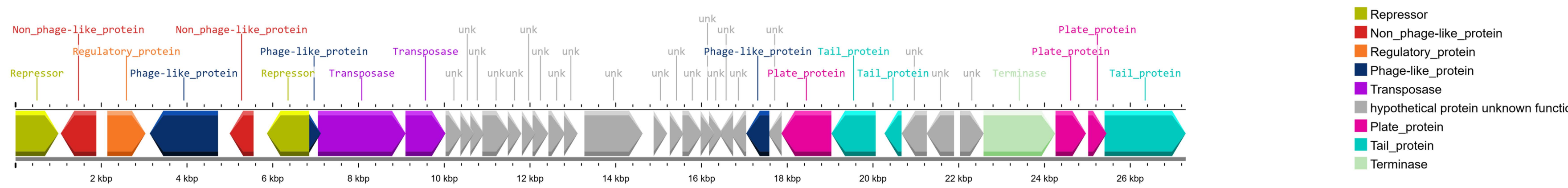

V.TUB.ph-33.2

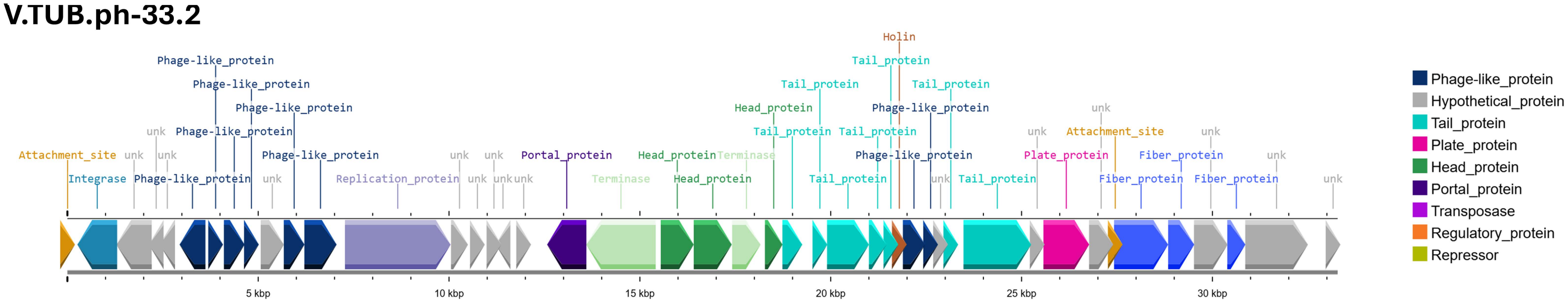

V.TUB.ph-36.5

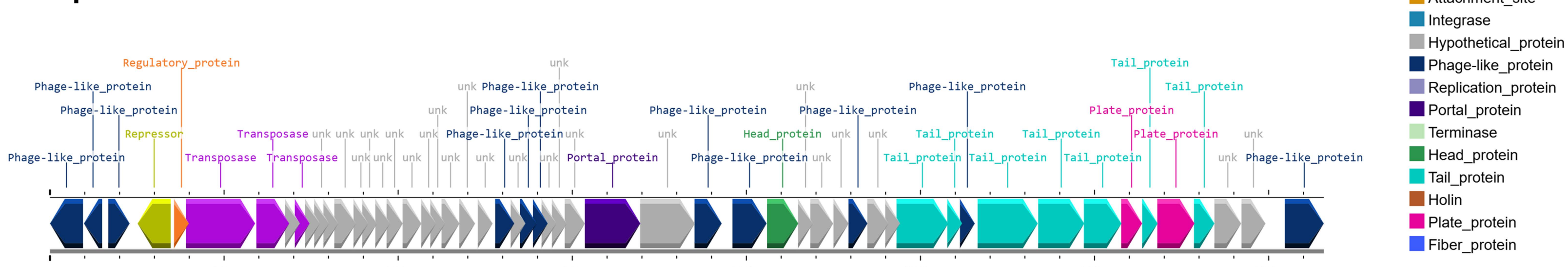

V.TUB.ph-36.6

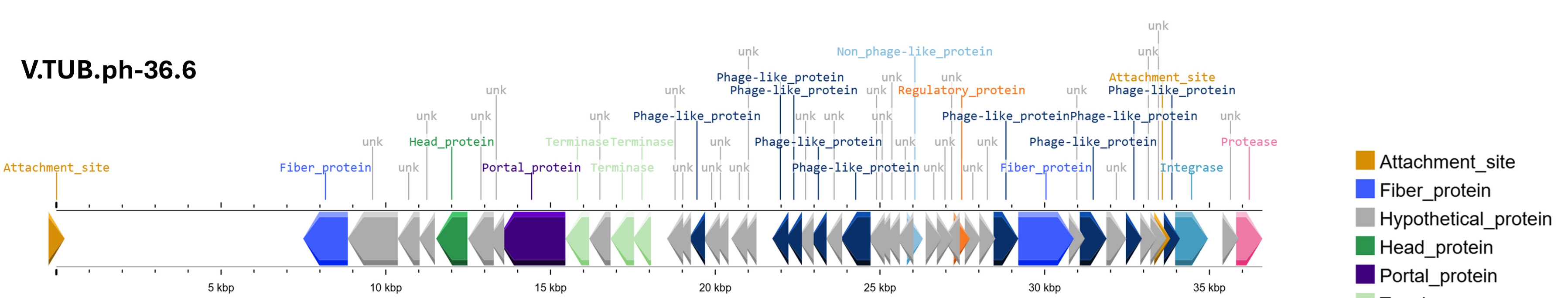

V.TUB.ph-40.0

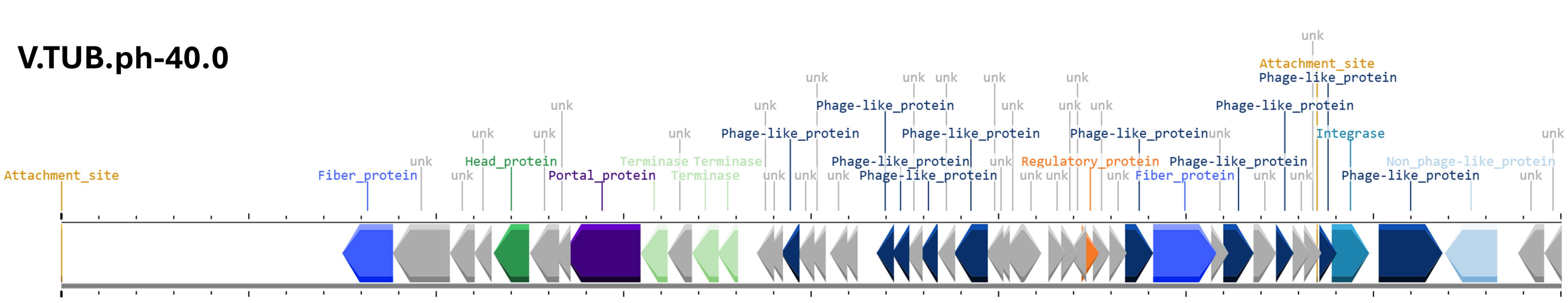

V.TUB.ph-73.8

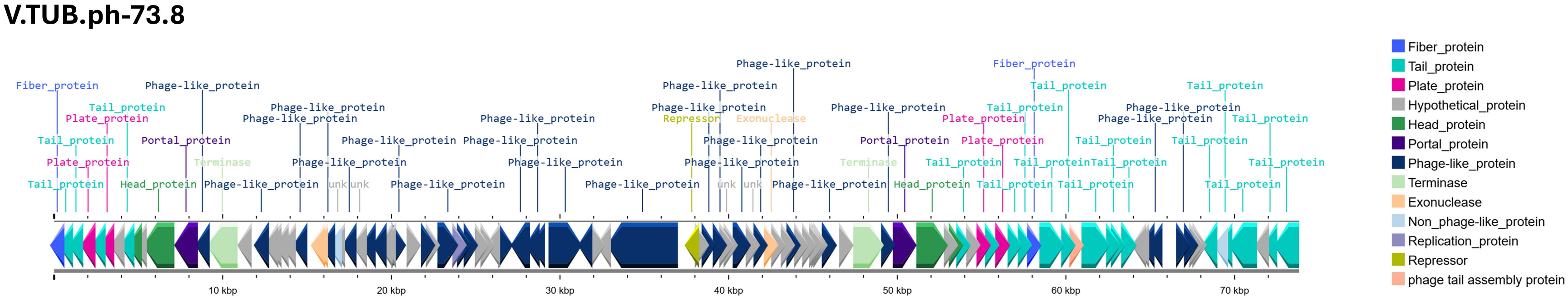

Supplementary Figure S5. Comparative circular alignment of plasmids from 3 *Vibrio tubiashii* strains, including two plasmids sequenced in this study (pVTU-TU1MA2-71 and pVTU-TU1MA1-47) and a reference plasmid from the NCBI database (pVTU-ATCC19109-57; see Table 3 for strain information and accession number). Each concentric ring represents a single plasmid and is labeled with its corresponding plasmid name. Colored features indicate functional gene categories, including conjugative transfer systems (brown), horizontal gene transfer–associated genes (pink), other functions (blue), and unknown functions (gray).

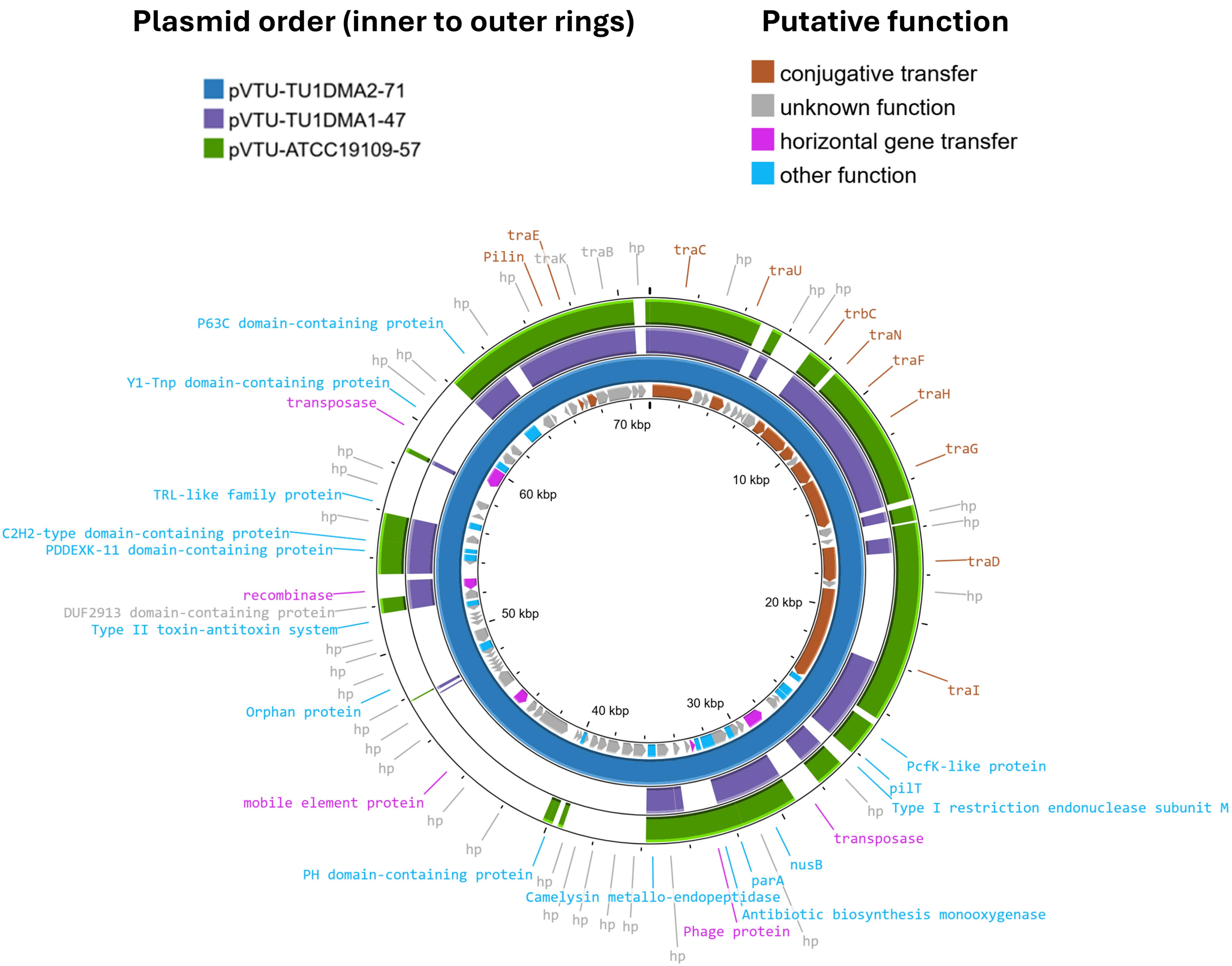

Plasmid order (inner to outer rings)
pVTU-TU1DMA2-71
pVTU-TU1DMA1-47
pVTU-ATCC19109-57
Putative function
conjugative transfer
unknown function
horizontal gene transfer
other function
70 kbp
10 kbp
20 kbp
30 kbp
40 kbp
50 kbp
60 kbp
traE
Pilin
traK
traB
hp
traC
traU
trbC
traN
traF
traH
traG
traD
traI
P63C domain-containing protein
Y1-Tnp domain-containing protein
transposase
TRL-like family protein
C2H2-type domain-containing protein
PDDEXK-11 domain-containing protein
recombinase
DUF2913 domain-containing protein
Type II toxin-antitoxin system
Orphan protein
mobile element protein
PH domain-containing protein
Camelysin metallo-endopeptidase
Phage protein
Antibiotic biosynthesis monooxygenase
parA
nusB
transposase
Type I restriction endonuclease subunit M
pilT
PcfK-like protein
